# Structure of the full-length human Pannexin1 channel and insights into its role in pyroptosis

**DOI:** 10.1038/s41421-021-00259-0

**Published:** 2021-05-04

**Authors:** Sensen Zhang, Baolei Yuan, Jordy Homing Lam, Jun Zhou, Xuan Zhou, Gerardo Ramos-Mandujano, Xueyuan Tian, Yang Liu, Renmin Han, Yu Li, Xin Gao, Mo Li, Maojun Yang

**Affiliations:** 1 Ministry of Education Key Laboratory of Protein Science, Tsinghua-Peking Center for Life Sciences, Beijing Advanced Innovation Center for Structural Biology, Beijing Frontier Research Center for Biological Structure, School of Life Sciences, Tsinghua University, Beijing 100084, China; 2 Laboratory of Stem Cell and Regeneration, Biological and Environmental Sciences and Engineering (BESE) Division, King Abdullah University of Science and Technology (KAUST), Thuwal 23955-6900, Kingdom of Saudi Arabia; 3 Computational Bioscience Research Center (CBRC), Computer, Electrical and Mathematical Sciences and Engineering (CEMSE) Division, King Abdullah University of Science and Technology (KAUST), Thuwal 23955-6900, Kingdom of Saudi Arabia; 4 Peking-Tsinghua Center for Life Sciences, Academy for Advanced Interdisciplinary Studies, Peking University, Beijing 100871, China; 5 School of Pharmacy, Tongji Medical College, Huazhong University of Science and Technology, Wuhan 430030, China; 6 Present Address: Bridge Institute, USC Michelson Center for Convergent Biosciences, University of Southern California, Los Angeles, CA 90089 USA

**Keywords:** Cryoelectron microscopy, Ion channel signalling

## Abstract

Pannexin1 (PANX1) is a large-pore ATP efflux channel with a broad distribution, which allows the exchange of molecules and ions smaller than 1 kDa between the cytoplasm and extracellular space. In this study, we show that in human macrophages PANX1 expression is upregulated by diverse stimuli that promote pyroptosis, which is reminiscent of the previously reported lipopolysaccharide-induced upregulation of PANX1 during inflammasome activation. To further elucidate the function of PANX1, we propose the full-length human Pannexin1 (hPANX1) model through cryo-electron microscopy (cryo-EM) and molecular dynamics (MD) simulation studies, establishing hPANX1 as a homo-heptamer and revealing that both the N-termini and C-termini protrude deeply into the channel pore funnel. MD simulations also elucidate key energetic features governing the channel that lay a foundation to understand the channel gating mechanism. Structural analyses, functional characterizations, and computational studies support the current hPANX1-MD model, suggesting the potential role of hPANX1 in pyroptosis during immune responses.

## Introduction

Pannexin is a family of large-pore forming channels that play pivotal roles in cell-to-cell communication. Among the three isoforms of Pannexin (PANX1, PANX2, and PANX3)^1^, PANX1 is ubiquitously expressed in mammalian cells and has been extensively studied for its diverse activation modes, association with diverse signaling molecules and broad cellular and tissue distribution^2^. The regulation of the PANX1 channel is complicated and remains unclear, but it is commonly accepted that PANX1 is regulated by extracellular potassium, intracellular calcium, ATP, mechanical stimuli, voltage, low oxygen stress, S-nitrosylation, and caspase cleavage^3–9^. PANX1 was shown to exhibit different conformations with different conductance and permeability properties depending on which type of stimulus resulted in channel activation^4,9–11^. The pore-associated C-termini inhibit the PANX1 channel function effectively^12,13^. However, it was also shown that caspase cleaved PANX1 or expression of PANX1 truncated at caspase cleavage site exhibits the same chloride selectivity as the wild type (WT) PANX1^10^, suggesting that C-termini might not be the exclusive factors responsible for these channel properties. One major hurdle in reconciling functional studies of PANX1 is the lack of structural insight of its C-terminus despite numerous attempts. Therefore, the structural and functional relationship of PANX1 is still open for investigation.

Although recent studies have provided important structural insights into the PANX1 protein^14–19^, the lack of the C-termini and/or N-termini in these models—potentially due to construct strategies (e.g., truncation of C-termini, tagging in C-termini)—have not fully elucidated the regulation mechanisms of PANX1 as both C-termini and N-termini were shown to inlay the channel pore in various experiments^12,13,20^. To better illustrate the function of PANX1, we designed a hPANX1 expression construct, with a Strep-tag inserted to the intracellular linker domain of hPANX1, to facilitate cryo-EM studies. In this respect, our models have revealed that both the N-termini and C-termini of PANX1 protrude deeply into the channel pore funnel, resulting in a tightly lined pore. MD simulations of hPANX1 also revealed a key features of ion permeation profile and channel-gating amino acids consistent with findings in a previous substituted cysteine accessibility method (SCAM) experiment^20^. Functional analyses of selected residues suggested by the structural model to be functionally important in human macrophages showed that S424 (channel pore residue) mutation promoted pyroptotic cell death, while N255 (glycosylation site) and D379 (caspase-cleavage site) mutations hindered pyroptotic cell death. Thus, these functional data support the current hPANX1 structural model and emphasize the role of hPANX1 in pyroptosis.

## Results

### Structure determination of the full-length hPANX1

A previous study suggested that the cleavage of PANX1 by caspase-11 triggers the efflux of cellular ATP to promote the P2X7-dependent pyroptosis^21^. The presence of C-termini signifies pre-activated states of pyroptosis^21^, but how does it maintain a close-state PANX1 channel is unclear. Using different construct tagging strategies (typically on C-termini with or without protease cleavage), several groups have reported the structures of the PANX1 channel in different states^14–19^, including the apo state, carbenoxolone-bound state, caspase-cleaved state, and gap-junction-like state. To approach efficient acquisition of full-length hPANX1 samples for structure determination, we expressed full-length hPANX1 together with a Strep-tag to allow purification via affinity chromatography. As both N-termini and C-termini of hPANX1 were proposed to inlay the channel and contribute to the channel function^13,20^, we avoided strategies that insert tags to either terminus. Instead, we conducted extensive experiments and ultimately identified that residue V172—present in the intracellular linker domain—was suitable for inclusion of a Strep-tag; this linker domain was shown outside of the pore in previous structural studies^14–19^.

**Fig. 1:**
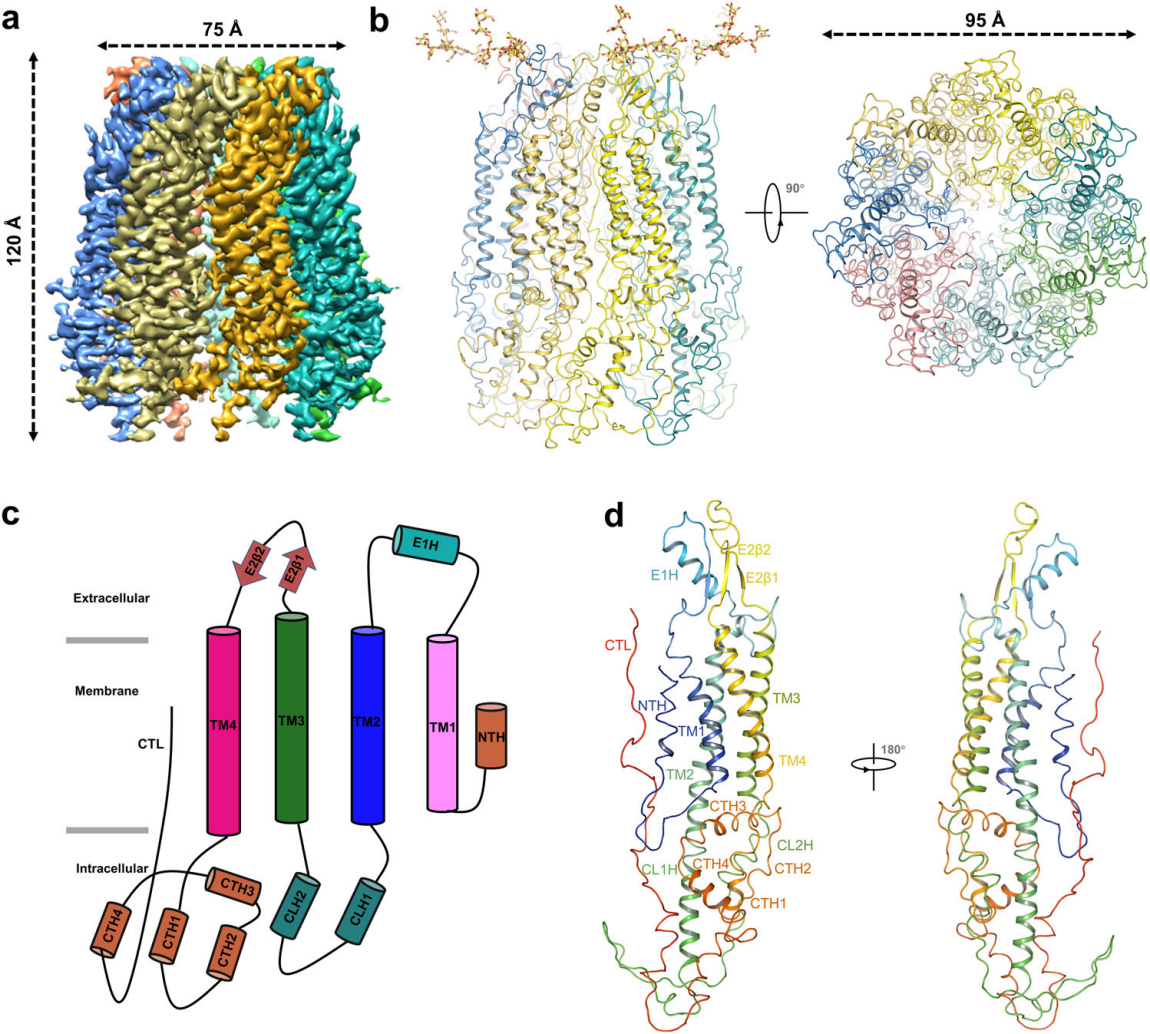
Cryo-EM structure of the hPANX1 channel. **a** Cryo-EM density map of hPANX1 at a resolution of 3.15 Å with each subunit color-coded from a side view. **b** Ribbon representation of the heptameric hPANX1-MD structure from the side view and the bottom view with each subunit color-coded. The glycosylation moieties are shown as sticks. **c** Schematic representation of the secondary structure of hPANX1. Cylinders and arrows indicate helices and β-strands, respectively. **d** Ribbon representation of the hPANX1-MD protomer structure with different regions colored.

Single-particle analysis produced a 3D reconstruction of hPANX1 resolved at 3.15 Å under the gold-standard Fourier shell correlation criteria (FSC = 0.143), which was of sufficient quality to build models for the majority of protein regions (Supplementary Figs. S1–S5 and Table S1). The final map yielded a heptameric hPANX1 channel with a volume of 95 × 75 × 120 Å (Fig. 1), and we constructed a raw hPANX1 model in accordance with the raw cryo-EM density map. However, despite the confirmed presence of the full-length hPANX1 protein in the cryo sample, the electron densities accounting for the intracellular link (160–194) between TM2 and TM3 and the C-termini were subjected to local density falloff without local scaling and sharpening. The resultant cryo-EM density after local scaling and sharpening also shows an outgrowth of density at the intracellular domain exterior of the pore when compared to maps obtained from the previous experiment; this provides evidence for the location of the Strep-tag (Supplementary Fig. S6). Nonetheless, similar to the reported PANX1 structures^14–18^, the seven subunits of our model were arranged around an axis of symmetry that defined an ion conduction path perpendicular to the membrane plane (Fig. 1a, b). The hPANX1 protomer resembles other gap junction proteins (Connexins and Innexins) and volume-regulated chloride channel (LRRC8A) (Supplementary Fig. S7). Specifically, hPANX1 contains short N terminal helices (NTH) facing the inner pore tunnel and stretching into the channel pore (Fig. 1c, d).

### N255 glycosylation regulates membrane trafficking of hPANX1

**Fig. 2:**
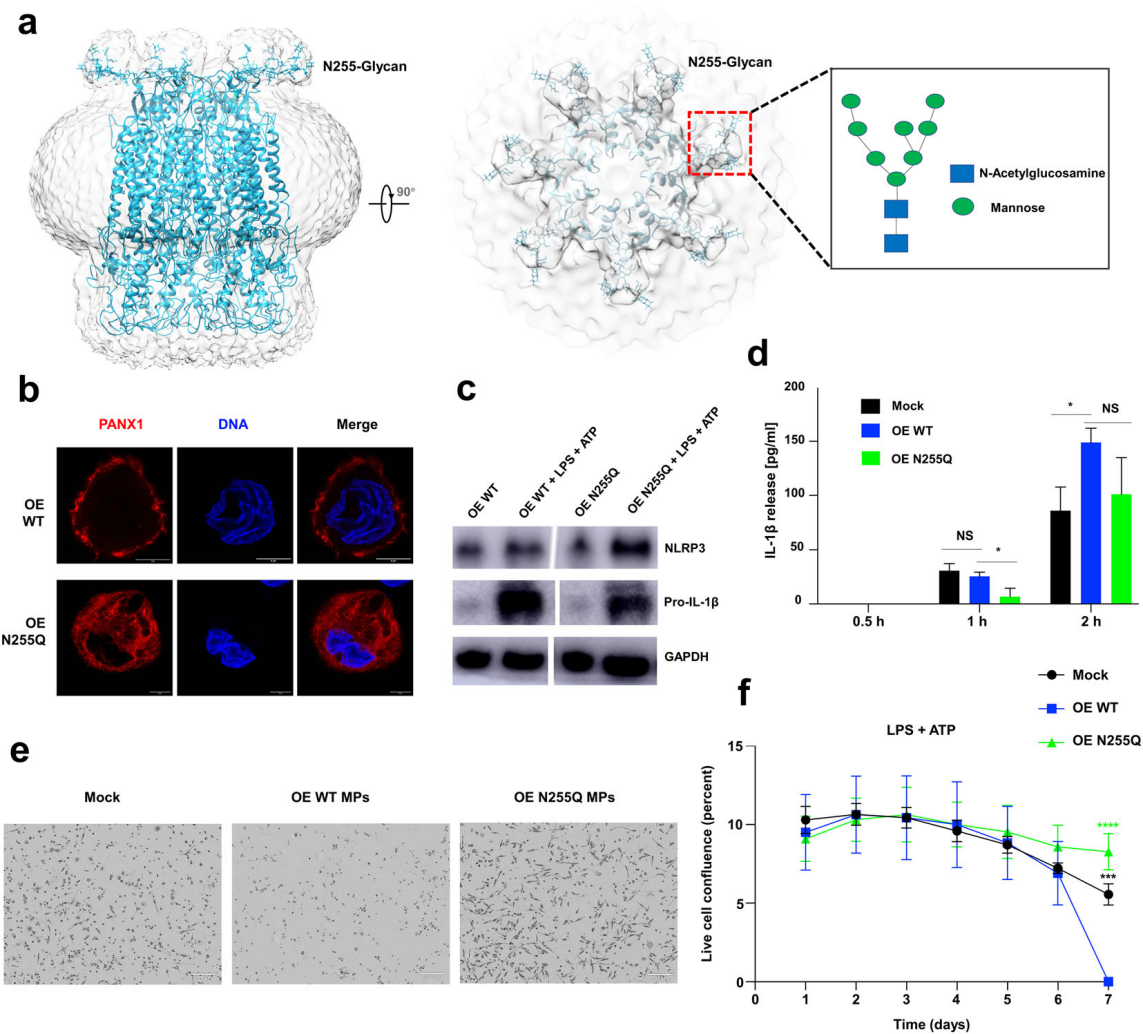
Structural and functional analysis of the glycosylation modification in hPANX1. **a** Schematic representation of hPANX1 heptamer in light blue docked into the cryo-EM density map. The middle panel shows the top view of hPANX1 with glycosylation indicated. The right panel shows a magnified view of the N-linked glycans (including two N-Acetylglucosamine and eight Mannose). **b** Representative images of the localization of WT and N255Q hPANX1 in THP-1-derived macrophages (MPs). The images were collected using the super-resolution STED mode. OE is the abbreviation of overexpression in all the figures. Scale bar = 5 µm. **c** Western blot analysis of the NLRP3 and Pro-IL-1β in WT hPANX1 overexpressing and N255Q hPANX1 overexpressing MPs. MPs were treated with LPS (1 µg/ml) and ATP (3 mM) for 24 h. GAPDH was used as a loading control. **d** IL-1β release from mock, WT hPANX1 overexpressing and N255Q hPANX1 overexpressing MPs. MPs were primed with LPS (1 µg/ml) overnight and stimulated with nigericin (10 µM) for 0.5, 1, or 2 h. IL-1β release was measured by ELISA. Error bars represent s.e.m. for *n* = 3. *P < 0.05, NS not significant (one-way ANOVA followed by Dunnett’s test, OE WT set as comparison control). **e** Phase-contrast images of MPs stimulated by LPS and ATP. Images were acquired with an IncusyteS3 system on day 6. **f** Time course of live cell confluence of MPs. Image data were collected every 2 h and analyzed with an IncusyteS3 system. Error bars represent s.d. for *n* = 4. ***P < 0.001, ****P < 0.0001 (one-way ANOVA followed by Dunnett’s test, OE WT set as comparison control).

Previous studies demonstrated that the degree of N-glycosylation is correlated with the subcellular localization and channel function of PANX1^22,23^. In our cryo-EM map, a significant outgrowth of electron density with an antenna-like topology at the residue N255 (Fig. 2a) suggests a physical barrier to prevent two PANX1 hemichannels from forming a gap junction channel^22,23^. Using SDS-PAGE, we identified three N-glycosylation forms of purified hPANX1, all of which were reduced to the non-glycosylation form either following PNGase F glycosidase treatment or by the mutation of N255Q (Supplementary Fig. S1a, b). Our results are consistent with previous studies of the glycosylation site (N254) in rat and mouse PANX1 (equivalent residue to human N255)^22,23^, including the glycosylation forms and subcellular localization, suggesting significant regulatory roles of N255 in membrane trafficking for hPANX1. The glycosylation content on N255 was further established as two N-acetylglucosamine and eight mannoses in a complex manner (Fig. 2a) by higher-energy collisional dissociation (HCD) and collision-induced dissociation (CID) mass spectrometry (Supplementary Fig. S8).

We next sought to construct a human cellular model to study the functional significance of the residues (e.g., N255) suggested by the structural model to be important for hPANX1 function. PANX1 is highly expressed in human and mouse monocyte and macrophage cell lines and is further upregulated after lipopolysaccharide (LPS) stimulation^24^. An analysis of published transcriptomic data in the Stemformatics database^25,26^ further supports significant upregulation of PANX1 in macrophages subjected to various immunological challenges^27–30^ (e.g., Toll-like receptor (TLR) agonist, proinflammatory cytokine, tuberculosis, Supplementary Fig. S9a). Thus, we decided to use macrophages differentiated from the human THP-1 monocytic cell line as a model to study hPANX1. We showed that hPANX1 was significantly upregulated at both mRNA and protein levels in THP-1-derived macrophages or in macrophages stimulated by TLR agonist (R848) or E. coli bioparticles (Supplementary Fig. S9b). PANX1 was shown to mediate several forms of cell death^5,21,31^, including a lytic pro-inflammatory type of cell death called pyroptosis, characterized by cell swelling, pore formation, and rapid destabilization of membrane integrity^32^. We next established hPANX1 knock-down THP-1 cell lines using two highly effective shRNAs at both the mRNA and protein levels (Supplementary Fig. S9c, d). Live cell imaging showed that THP-1-derived macrophages underwent cell death through massive swelling and rupture of cell membrane characteristic of pyroptosis upon various stimulations (e.g., purinergic P2X7 receptor agonist, TLR agonist R848, nigericin, and *E. coli* bioparticles) (Supplementary Fig. S10 and Movies 1–9). Importantly, knockdown studies showed that these biological processes depended on the presence of hPANX1 (Supplementary Fig. S10). Therefore, we concluded that THP-1-derived macrophages were a suitable human cellular model for functional dissection of hPANX1 especially in pyroptosis.

We constructed an inducible system for overexpression of WT hPANX1 and hPANX1 N255Q mutant (hereafter referred to as N255Q, Supplementary Fig. S11a–c) in THP-1 cells to validate the functional significance of N255 in pyroptosis. N255Q only had the Gly0 form, consistent with our in vitro data (Supplementary Figs. S1b and S11c). Stimulated emission depletion (STED) super-resolution microscopy revealed that the endogenous and overexpressed WT hPANX1 correctly localized to the plasma membrane^22^ (Fig. 2b and Supplementary Fig. S11d), whereas N255Q was predominantly located to the cytoplasm (Fig. 2b). We next investigated the effect of the mutation on intracellular levels of NLRP3 and IL-1β during pyroptosis of macrophages. WT hPANX1 overexpressing cells recapitulated the upregulation of NLRP3 and pro-IL-1β upon LPS priming and ATP stimulation as seen in cells expressing the endogenous hPANX1 (Fig. 2c and Supplementary Fig. S11e). Meanwhile, higher levels of WT hPANX1 resulted in more IL-1β release and faster pyroptotic cell death than the endogenous level (Fig. 2d–f). Overexpression of N255Q led to higher levels of NLRP3 but lower levels of pro-IL-1β than WT hPANX1 following LPS priming and ATP stimulation (Fig. 2c). Consistently, the early release of mature IL-1β decreased significantly in N255Q macrophages, but no statistically significant difference was observed at a later time point (Fig. 2d). The reduction in IL-1β release in N255Q coincided with an attenuated pyroptotic cell death induced by LPS and ATP (Fig. 2e, f).

### De novo modeling reveals the pore funnel structure of hPANX1

PANX1 was suggested to contain a peculiar pore structure^20^. By analyzing inhibitory effects of thiol reagents on PANX1 with SCAM, it was shown that a part of TM1 and the first extracellular loop are located in the outer part of the pore, while the inner part of the pore is lined by the C-termini^20^ rather than the N-termini as in connexins^33^. Subsequent patch-clamp experiments further demonstrated that C-termini can contribute to strong autoinhibitory effects, which can be lifted naturally in apoptosis when C-termini are cleaved by caspase-3/7 at residue position 376–379 (site B)^5^. In addition, a previous study indicated that the mutated residue F54C—located in the extracellular end of the TM1—could form a disulfide bond with the terminal cysteine (C426)^12^. All these observations suggest the location and critical roles of the C-termini in PANX1 channel gating. In our raw cryo-EM map for hPANX1, the density accounting for the inner layer of the channel, though present, was subjected to local resolution-dependent amplitude falloff, which impeded all-atom modeling of C-termini due to the lack of necessary contrast in density. To amplify the contrast and to accurately characterize the pore funnel, we sharpened the map with the Local Scaling algorithm^34^ to produce a LocScale map. Subsequently, to verify main-chain features in the sharpened region, a mean-shift algorithm^35,36^ was applied to visualize densities as local dense points (LDPs) and the all-atom structure was interactively modeled by following closely to the LDP trails (Supplementary Fig. S12 and Movie S10). The final phase of the atomic modeling was completed with rounds of Cascade Molecular Dynamics Flexible Fitting (cMDFF)^37,38^ and Real-Space Refinement (RSR) of Phenix (Supplementary Fig. S12a, b). We tentatively proposed the final structure as the hPANX1-MD model, which was generated based on the LocScale map and cMDFF-RSR modeling. The density of the flexible CTD has been vastly improved after Local scaling and sharpening algorithm (Supplementary Fig. S13). As the eight-residue Strep-tag (WSHPQFEK) was located after residue V172, in the following paragraphs, we supplemented the native residue indexing with respect to the model in parentheses.

**Fig. 3:**
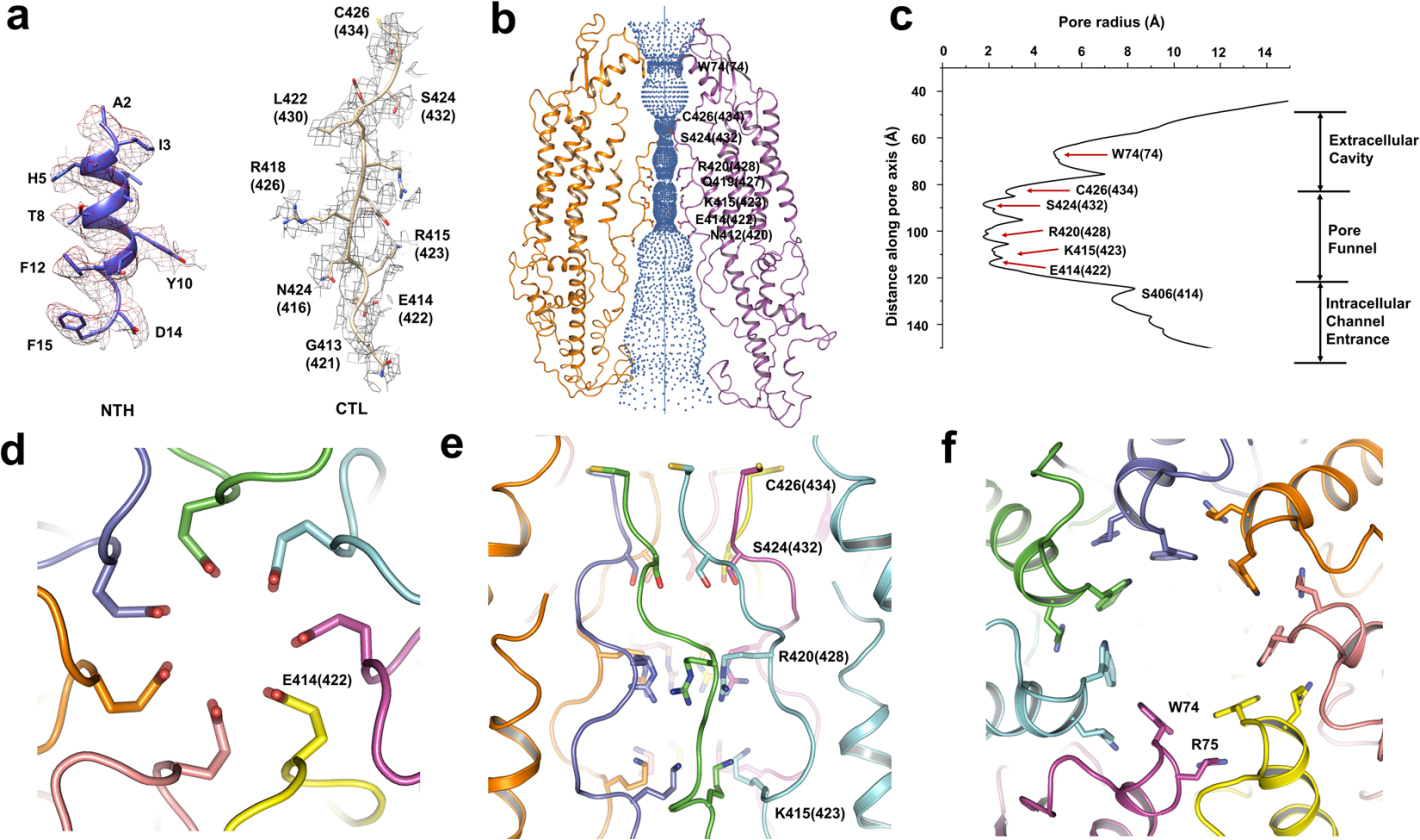
Ion permeation pathway of hPANX1. **a** The EM density of the N terminal helix (2–15) and the C terminal loop (412(420)–426(434)) with a contour level of 4σ. **b** Ion permeation pathway of hPANX1 is shown along two diagonally opposed promoters using HOLE program. Ion permeation pathway contains the extracellular cavity, pore funnel, and intracellular channel entrance. W74(74), C426(434), S424(432), R420(428), Q419(427), K415(423), E414(422), and N412(420) are shown with side chains to compose the representative gating sites. **c** Pore radius of the hPANX1 channel pore calculated with program HOLE. **d** Ribbon representation of E414(422) with each protomer color-coded. E414(422) forms a major constriction with a pore radius around 2 Å in the channel. **e** Ribbon representation of the C-termini of hPANX1 with each protomer color-coded. Moderate constrictions amino acids (K415(423), R420(428), S424(432), and C426(434)) are shown with side chains along the pore funnel. **f** Ribbon representation of the extracellular cavity of hPANX1 with each protomer color-coded. W74 and R75 are shown with side chains along the extracellular cavity.

In the resultant full-length hPANX1-MD model, the permeation pathway of the hPANX1 channel consists of an intracellular channel entrance, a pore funnel, and an extracellular cavity (Fig. 3). The heptameric C-termini protrude deeply into the pore funnel and occupy the innermost layer (Fig. 3a, b). In particular, E414(422) and S424(432) formed major constrictions in the channel at the intra-cellular and extra-cellular ends respectively (Fig. 3b–e); the intracellular constriction was abruptly enlarged at N412(420) through a turn at G413(421) (Fig. 3b). This corroborates with previous SCAM experiments in that thiol modification of positions E414 and S424 produced the most significant inhibitory effects among all residues in mouse PANX1, whereas inhibitions were attenuated at positions N411, N412, and G413^20^. The location of C426(434) (Fig. 3e) is congruent with a previous study where this C-terminal cysteine was shown to be responsible for the irreversible thiol reaction^20^, as C426(434) locates in the inner layer of the pore funnel and is essential to channel conductance. In particular, W74 and R75, located at the beginning of the extra-cellular cavity (Fig. 3f), were proposed to be critical for ATP inhibition of hPANX1 currents^39^ and were essential to PANX1 ion selectivity^14^. Of residues mentioned above, W74, R75, N411, N412, G413, E414, K415, R420, S424, and C426 are conserved among most mammals (Supplementary Fig. S14).

### Energetics of ion permeation and selectivity

**Fig. 4:**
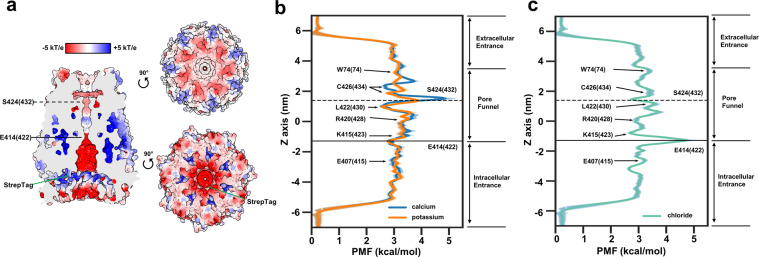
Energetics of ion permeation analysis of key residues in hPANX1 structure. **a** Surface representation of the full-length hPANX1-MD structure colored by coulombic potential (red, negative; white, neutral; blue, positive). Cross-section is colored in gray. *T* temperature, *k* Boltzmann constant, *e* charge of an electron. Location of Strep-tag (residue 173–180) is indicated by a green pointer. **b** Potential of mean force (PMF) profile describing the free-energy landscape in kcal/mol experienced by positive (potassium K^+^, orange trace; calcium Ca^2+^, blue trace) ions permeating the channel pore. Amino acid positions are indicated with arrows. E414(422) and S424(432) are marked on *Z*-axis as solid and dashed lines respectively. **c** Potential of mean force PMF profile describing the free-energy landscape in kcal/mol experienced by negative (chloride Cl^−^, green trace) ions permeating the channel pore. Amino acid positions are indicated with arrows. E414(422) and S424(432) are marked on *Z*-axis as solid and dashed lines respectively.

PANX1 was shown to exhibit low permeability for chloride/cation and virtually no permeability for ATP/small-molecule^4,9,10^ in its closed state. These properties had been attributed to autoinhibitory steric configurations in the C-terminus^12,13^. In agreement with these observations, our hPANX1-MD model has revealed numerous constrictions (Fig. 3b, c), especially those formed by E414(422) and S424(432) with pore radii that are narrower than the hydrated radius of common ions, such as calcium (Ca^2+^, 4.12 Å), chloride (Cl^–^, 3.32 Å), and potassium (K^+^, 3.31 Å). Thus, our current hPANX1 model is in a closed state. Given that the radius of Cl^-^ is 1.21 Å (Stokes) and 3.32 Å (hydrated), it seems that a minor conformational change in the C-terminus of PANX1 would suffice for the channel to adopt the chloride selective conformation. Consistent with such a hypothesis is the observation that C426 can be reacted with thiol reagents in the voltage-activated channel^20^. In particular, using Adaptive Poisson–Boltzmann Solver 1.3 (Fig. 4a), we showed that the intracellular entrance, paved with conserved negatively-charged sidechains (e.g., E19(19)/E22(22)/E407(415)), was guarded by a major constriction at E414(422), also negatively-charged. In the pore funnel region, the charge environment varies as it is paved with hydrogen-bond donor/acceptor (Q419(427), S424(432)) together with less exposed positively-charged sidechains (K415(423), R420(428)) (Fig. 3b, e).

To elucidate the effect of these complicated electrostatics as well as molecular motions in ion permeation processes, we have performed all-atom MD simulations to profile the potential-of-mean-force (PMF) for permeation of K^+^, Ca^2+^, and Cl^−^ ions (Fig. 4b, c and Supplementary Fig. S15), as these ions are related to PANX1 regulation^3,6,40,41^. The MD simulations were conducted on the hPANX1-MD model with the Strep-tag removed from the experimental model to prevent Strep-tag residues from blocking the intracellular entrance (Fig. 4a). In agreement with the low conductance observed experimentally, the majority of the ion passageway in hPANX1, irrespective to the identity of ion, is lined with energetic barriers of over 2.9 kcal/mol, which are relatively high in comparison to peak barriers (~2–3 kcal/mol) presented in simulations for connexins and other high-conductance sodium or potassium channels. The peak barriers for hPANX1 are even higher. For positive ions, the peak barrier (Ca^2+^ around 5 kcal/mol; K^+^ around 4 kcal/mol) is located around S424(432)—the major pore-funnel constriction (Fig. 4b); for Cl^–^, the peak barrier (around 5 kcal/mol) is located around E414(422)—the major intra-vestibule constriction (Fig. 4c). These results therefore corroborate with the position of charged residues and constrictions, supporting the importance of steric and electrostatic configurations of the C-terminus in controlling the conductance of hPANX1 in its inactive state^5,12,13,20^. In the current model, the channel widens a lot prior to S406(414) and those upstream regions of the C-termini (370(378)-406(414)) are subjected to higher fluctuations than the rest of the protein (Fig. 3c). This observation helps to reconcile with a seemingly contradictory claim about non-specific interaction between C-termini, and the rest of the protein^13^ in that YoPro influx is detectable only when the C-termini were truncated from residue index < 407.

**Fig. 5:**
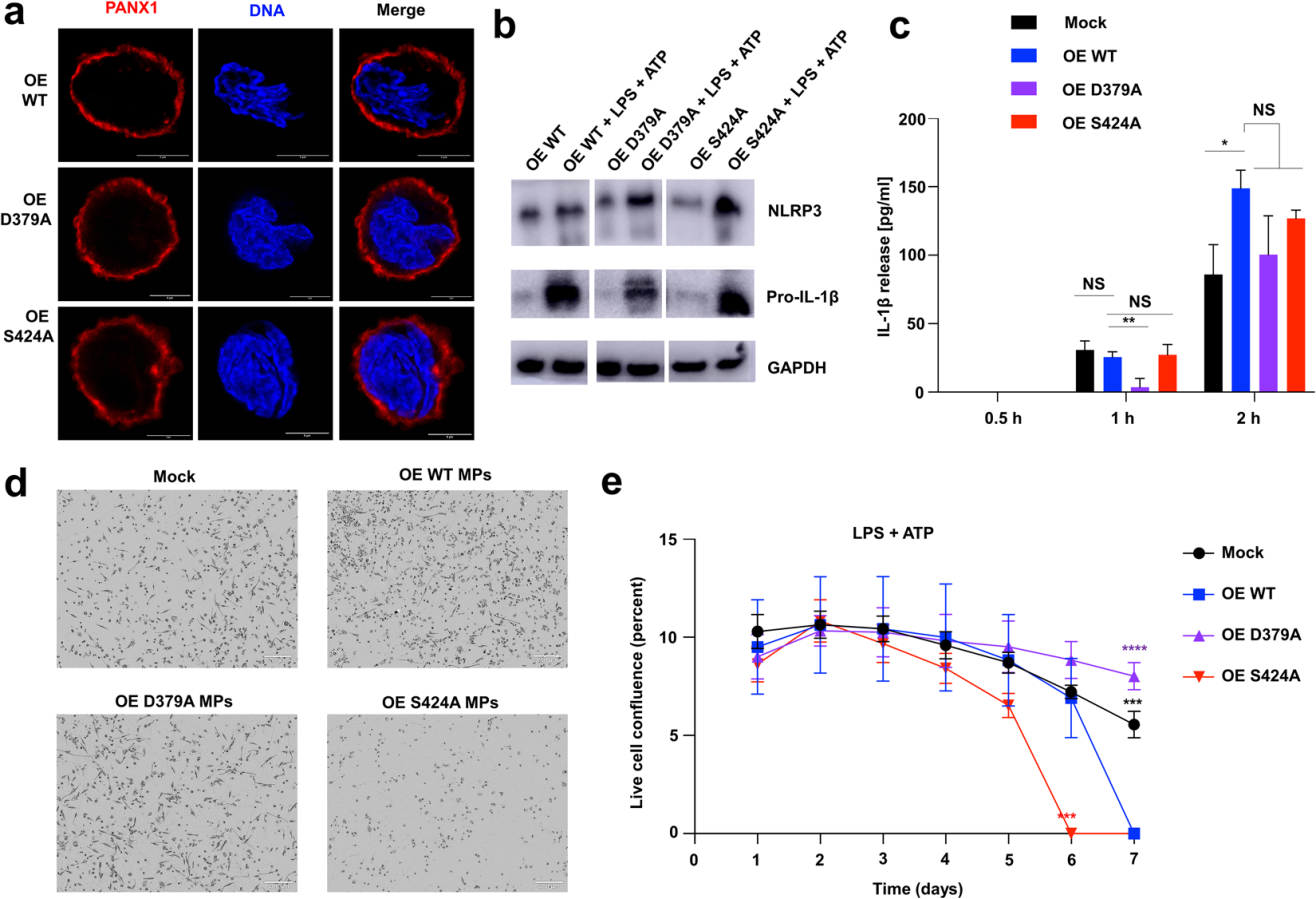
Cellular localization and pyroptosis-related functional analysis of D379A and S424A in macrophages. **a** Representative images of the localization of WT and mutant hPANX1 (D379A and S424A) in MPs. The images were collected using the super-resolution STED mode. Scale bar = 5 µm. **b** Western blot analysis of the NLRP3, Pro-IL-1β in WT and mutant (D379A and S424A) hPANX1 overexpressing MPs. MPs were treated with LPS (1 µg/ml) and ATP (3 mM) for 24 h. GAPDH was used as a loading control. **c** IL-1β release from mock, WT overexpressing and mutant (D379A and S424A) overexpressing MPs. MPs were primed with LPS (1 µg/ml) overnight and stimulated with nigericin (10 µM) for 0.5, 1, or 2 h. IL-1β release was measured by ELISA. Error bars represent s.e.m. for *n* = 3. *P < 0.05, **P < 0.01, NS not significant (one-way ANOVA followed by Dunnett’s test, OE WT set as comparison control). **d** Phase-contrast images of MPs stimulated by LPS and ATP. Images were acquired with an IncusyteS3 system on day 6. **e** Time course of live cell confluence of MPs. Image data were collected every 2 h and analyzed with an IncusyteS3 system. Error bars represent s.d. for *n* = 4. ***P < 0.001, ****P < 0.0001 (one-way ANOVA followed by Dunnett’s test, OE WT set as comparison control).

To further analyze the function of C-terminus of hPANX1 during pyroptosis in macrophages, we separately generated inducible THP1 cell lines that overexpress two hPANX1 mutants (D379A and S424A, Supplementary Fig. S11a–c), respectively. These mutants shared a similar level of glycosylation pattern and expressed a comparable level of protein with the WT PANX1 (Supplementary Fig. S11c). In addition, the same plasma membrane localization was observed in D379A and S424A compared with WT hPANX1 (Fig. 5a). We next investigated the effect of the mutations on intracellular levels of NLRP3 and IL-1β during pyroptosis of macrophages. However, whilst D379A and S424A only slightly increased NLRP3 levels, D379A decreased pro-IL-1β production following LPS priming and ATP stimulation (Fig. 5b). As with N255Q (Fig. 2d), D379A significantly decreased the early release of mature IL-1β, but not at a later time point (Fig. 5c). Interestingly, S424A promoted pyroptotic cell death whereas D379A attenuated cell death under the same condition (Fig. 5d, e). Together, these data provide functional evidence that the structurally informed residues (D379 and S424) are important for the function of hPANX1 in pyroptosis of human macrophages.

## Discussion

The current hPANX1-MD model establishes a homo-heptameric architecture of a large pore channel. A strategically designed Strep-tag was introduced after residue V172 to maintain channel pore integrity in which the N-terminus and C-terminus stretch into the inner pore funnel and interact with each other to block the large channel pore (Fig. 3a–c). Hence, the location of the affinity tag is a key determinant to the assembly of the homo-heptamer, as the constructs with the N-terminal or C-terminal affinity tag deform the circle-shaped heptamer and disrupt the channel pore integrity, triggered by in vitro affinity chromatography force stimuli during protein purification. The current proposed hPANX1-MD model is consistent with previous biological studies^5,12,13,20^ and provides significant insights into the functional mechanism of hPANX1. Though the current proposed model is supported by cryo-EM experiments and MD simulations, the densities accounting for the C-termini and N-termini are weaker than other domains in hPanx1, suggesting inhomogeneity of the native conformations. Therefore, additional studies to illustrate the regulatory mechanism of PANX1 should focus on purification strategies that further lessen artificial stress on the termini, for instance, development of specific antibody, in order to capture native functional states of PANX1.

Out of the three residues analyzed in the human THP-1-derived macrophage model, N255 (N254 in mice) and D379 have been shown to be an important for subcellular localization and catalytic activation of PANX1^5,22,23^. Mutation at these residues inhibited early IL-1β release, but not the late IL-1β release (Figs. 2d and 5c), which is in line with a previous finding that PANX1 was required for early IL-1β release at 2-h and 8-h post-stimulation with DOTAP/LPS or CTB/LPS^21^. However, one study found that the IL-1β and IL-18 were secreted normally in LPS-primed PANX1^−/−^ macrophages derived from mouse bone marrow^42^. It is possible that overexpression of these defective channels has dominant-negative effects on the formation of large membrane pores (e.g., sequestration of protein effectors, such as Gasdermin D^43^), that are responsible for IL-1β release. S424 is a conserved major pore-funnel constriction site and a peak barrier to cations in hPANX1 (Fig. 4b and Supplementary Fig. S14). The impact of the S424A mutation is not expected to manifest in scenarios in which C-termini of hPANX1 is cleaved at the canonical caspase site D379 followed by rapid cell death. Our nigericin stimulation results are consistent with this idea (Fig. 5c). Interestingly, when macrophages were stimulated by LPS and ATP, where cells die more gradually, the S424A mutation promoted cell death (Fig. 5d, e). Although the detailed mechanism awaits further study, one possibility is that S424A disrupts the cation barrier of the hPANX1 channel (Fig. 4b), resulting in increased permeability of ions such as K^+^. This in turn may activate hPANX1 channels and promote ATP release^3,44,45^, thus leading to the accelerated cell death in the S424A mutant. Moreover, the decrease of cytosolic K^+^ is a common step that is necessary and sufficient to activate the NLRP3 inflammasome^46^, which offers another explanation for the accelerated cell death that is independent of the P2X7 pathway.

Notably, from our subsequent MD analysis on the PMFs of positive ions, the global maxima at S424(432) was sandwiched between electronegative terminal carboxyl of C426(434) and a hydrophobic L422(430), suggesting that the presence of these residues may play roles to rarefy the passage of positive ions by attracting the positive ions to C426(434) or repelling the hydrated ions at L422(430) from the intracellular side. In particular, the terminal carboxyl of C426(434) appears to adopt asymmetric heptameric configurations, which are occasionally stabilized by S425(433) and the cysteine side chain to accommodate potassium ions (Supplementary Fig. S15h). S425(433) and C426(434) are remarkably conserved among species (Supplementary Fig. S14). However, considering the sub-microsecond timescale of our simulations, it is unclear if these concerted movements of C-termini are relevant to the experimental activation by extracellular potassium^3,4,44,45^.

Overall, the diameter of the extracellular gate in the open-state PANX1 is around 1 nm^16^, which is not wide enough to translocate the inflammatory cytokines, such as IL-18. Thus, it is plausible that hPANX1 is not directly involved in the release process of inflammatory cytokines. The well-established form of pyroptosis is mediated via the cleavage of Gasdermin D by caspase-1/4/5/11, thereby priming the release of inflammatory cytokines. It remains an open question whether the PANX1-P2X7-mediated pyroptosis represents an independent form of pyroptosis or cooperates with Gasdermin mediated pyroptosis. Our mutagenesis assays (Figs. 2d and 5c) indicated that hPANX1 might function as an upstream signal mediator of Gasdermin D, as the early release of mature IL-1β was significantly decreased in N255Q and D379A mutants compared with WT, although the precise role of hPANX1 in pyroptosis needs further investigations.

Interestingly, STED super-resolution imaging revealed that hPANX1 channels localize to puncta 100–300 nm in diameter (Supplementary Fig. S11d), which is intuitively larger than the heptamer structure (12 nm in diameter, Fig. 1a, b). Such a puncta localization in the plasma membrane has been observed in previous studies among different cells^47–50^, and a remarkable increase of the puncta was detected after LPS stimulation^50^. Moreover, hPANX1 was suggested to be part of the pore-forming unit of the P2X7 receptor death complex in the *Xenopus oocyte* heterologous expression system^51^. It is plausible that this form of puncta localization is attributed to the aggregation of hPANX1 proteins to interact with other proteins^52^, such as ion channels, receptors, and their signaling complexes. Further evaluation should probably await the analysis of the hPANX1 interacting signaling complex during immune response through the cryo-electron tomography (cryo-ET).

## Materials and methods

### Cell culture and transfection

For protein expression, *Homo sapiens* PANX1 was cloned in frame with a Strep-tag II (WSHPQFEK) into the plasmid pcDNA3.1(−) and the Strep-tag was located after amino acid V172 after sufficient test. HEK 293F cells (Invitrogen) were cultured in SMM 293T-I medium (Sino Biological Inc.) under 8% CO_2_ in a Mulititron-Pro Shaker (Infors, 120 rpm) at 37 °C. Transient transfection was performed to heterogeneously express the target protein. In brief, for 1-l culture of HEK293F cells, 1 mg plasmid was pre-incubated with 4 mg 25-kDa linear polyethylenimines (PEIs) (Polysciences) in 50 ml fresh medium for 30 min prior to adding the mixture to cells. The transfected cells were cultured for 48 h before harvest.

THP-1 cells were cultured in the RPMI-1640 medium (Thermo Fisher Scientific, 21875034) supplemented with 0.05 mM 2-mercaptoethanol (Thermo Fisher Scientific, 21985–023) and 10% heat-inactivated fetal bovine serum (Thermo Fisher Scientific, 16140071). To differentiate THP-1 cells into macrophages (MPs), 50 ng/ml phorbol 12-myristate 13-acetate (PMA, Sigma-Aldrich, P1585–1MG) was added to the growth medium and maintained for 24 or 48 h. THP-1 derived MPs were stimulated for the indicated time with 30 µM R848, pHrodo E. coli bioparticles (Life Technologies, P35361), 1 μg/ml LPS (Novus Biologicals, NBP2-25295), R848 plus bioparticles, LPS plus 3 mM ATP (Sigma-Aldrich, A2383-5G), or primed with LPS and stimulated with 10 µM nigericin (MERCK Sigma-Aldrich, 481990). Cells were maintained in humidified incubators at 37 °C under 5% CO_2_.

### Live cell microscopy

THP-1 cells were seeded at 1 × 10^5^ per well in 24-well tissue culture plates (Corning, 353047) followed by 2-day macrophage differentiation induced by PMA (50 ng/ml). The cells were treated with stimuli before imaging. An IncucyteS3 equipment was used to collect real-time live images with four images per well in two technical replicates. The image data were analyzed using the IncucyteS3 basic software. All live images were acquired using a 10× objective lens. The acquisition time of the red channel was 400 ms.

### Confocal microscopy

Cells were seeded in 8-chamber slides and fixed with 4% formaldehyde in PBS for 30 min at room temperature. Subsequently, the cells were permeabilized with 0.2% Triton X-100 and blocked with 6% normal donkey serum in PBS for 1 h at room temperature. Then the cells were incubated with PANX1 (D9M1C) rabbit mAb (Cell Signaling Technology, 91137 S) at 4 °C overnight, followed by goat anti-rabbit IgG Alexa Fluor ^®^594 (Abcam, ab150080) incubation at room temperature for 1 h. Nuclei were counterstained with Picogreen (Life Technologies, P7581) and mounted with ProLong^TM^ Gold (Life Technologies, P36934). Images were acquired with a Leica SP8 TCS STED 3× microscope.

### Protein analysis by immunoblotting

Protein samples were isolated and further separated by 4%–12% SDS-PAGE gel (NW04125BOX, Thermo Fisher Scientific). After protein transfer, immunoblots were performed using specific primary antibodies and HRP-conjugated secondary antibodies. Primary antibodies are as follows: pannexin-1 (D9M1C) rabbit mAb (91137S, 1:1000, Cell Signaling Technology), anti-NLRP3/NALP3, mAb (Cryo-2) (AG-20B-0014-C100, 1:1000, AdipoGen), IL-1β polyclonal antibody (PA420B, 1:1000, Invitrogen), cyclophilin B polyclonal antibody (PA1027A, 1:2000, Invitrogen), and GAPDH antibody (MA5-15738, 1:2000, Invitrogen). Secondary antibodies are as follows: donkey anti-mouse IgG secondary antibody (SA1-100, 1:2000, Thermo Fisher Scientific) and donkey anti-rabbit IgG secondary antibody (SA1-200, 1:2000, Thermo Fisher Scientific). Signals were detected with ECL Western Blotting Substrate (32106, Thermo Fisher Scientific).

### Detection of human IL-1β release

PANX1 overexpressing cell lines were cultured in 96-well plates (20,000 cells/well), induced to MPs and treated with doxycycline (Dox, 2 µg/ml, 48 h). All cell lines were primed with LPS (overnight) and incubated with 10 μM nigericin for 0.5, 1, and 2 h. hIL-1β release was quantified using 100 µl of culture supernatant by enzyme-linked immunosorbent assay (ELISA) using the Mini TMB ELISA Development Kit (PEPROTECH 900-TM95) according to the manufacturer’s protocol; then, 1-Step™ Ultra TMB-ELISA (Thermo Scientific; 34028) was added (15–30 min), and the reaction was stopped by the addition of 2 M sulfuric acid. The absorbance was measured at 450 nm using a plate reader (BioTek, Synergy H1). Standard curve was included to obtain the hIL-1β concentration in the samples.

### Molecular cloning

Vectors containing shRNAs that target hPANX1 were purchased from Sigma Aldrich. The WT hPANX1 coding sequence was cloned from cDNA of THP-1 cells and confirmed with Sanger sequencing. Point mutations were introduced to the WT hPANX1 by direct PCR or assembly PCR. The WT and mutant hPANX1 fragments were inserted into the pInducer20 plasmid (Addgene, #44012) using Gateway cloning. The shRNA vectors and hPANX1 vectors were used to produce lentiviruses using the 3rd generation and 2nd generation lentiviral packaging system following standard protocols, respectively. Briefly, HEK 293T cells with above 90% confluence were transfected using lipofectamine 3000 reagent (Invitrogen, L3000001) premixed with vectors of pMDLg/pRRE (Addgene, #12251), pRSV-Rev (Addgene, #12253), pMD2.G (Addgene, #12259) and the corresponding shRNA vector, or using the TransIT®-Lenti Transfection Reagent (Mirus Mirus Bio, MIR6604) premixed with plasmids of psPAX2 (Addgene, #12260), pMD2.G and pInducer20 reconstructed with WT or mutant (N255Q, D379A or S424A) hPANX1. The lentiviruses were collected 48 h post-transfection, filtered through a 0.45-µM filter, and concentrated using the PEG-IT™ virus precipitation solution (System Biosciences, LV810A-1). THP-1 cells were transduced with each concentrated lentivirus three times and selected in the presence of 1 µg/ml puromycin or 0.5 mg/ml G418. After selection, total RNA was isolated using TRlzol™ Reagent and hPANX1 mRNA level was determined by qRT-PCR in each generated cell line.

### Phagocytosis assay

THP-1 cells were seeded at 1 × 10^5^ per well in 24-well tissue culture plates (Corning, 353047). The medium was changed after a two-day macrophage differentiation induced by PMA (50 ng/ml). The pHrodo^TM^ red bioparticles opsonized by opsonizing reagents (Life Technologies, E2870) were premixed with 30 µM R848 or added into the wells directly at a final concentration of 20 µg/ml. The images were collected immediately using an IncucyteS3 equipment every hour.

### Protein expression and purification

For each batch of protein purification, two liters of transfected HEK293F cells were harvested by centrifugation at 3000× *g*. Cell pellets were resuspended in the lysis buffer containing 20 mM Hepes, pH 7.4, and 150 mM NaCl, 1 μg/ml leupeptin, 1.5 μg/ml pepstatin, 0.84 μg/ml aprotinin, 0.3 mM PMSF, and lysed by sonication for 5 min. The cell membrane was pelleted after a 100,000× *g* ultracentrifugation for 1 h. The membrane was resuspended in the buffer containing 20 mM Hepes, pH 7.4, 150 mM NaCl, 2 mM DTT, and 1% (w/v) C12E9 for 2 h with gentle rotation at 4 °C. After ultra-centrifugation at 100,000× *g* for 20 min, the supernatant was incubated with Strep-Tactin Sepharose (IBA) for 1 h with gentle rotation at 4 °C. The resin was washed extensively with the wash buffer containing 20 mM Hepes, pH 7.4, 150 mM NaCl, and 0.1% digitonin. The target hPANX1 protein was eluted with wash buffer plus 5 mM d-Desthiobiotin (IBA) and concentrated to a final volume of approximately 100 μl. The final protein was applied to size-exclusion chromatography (Superpose-6 10/300 GL, GE Healthcare) in the buffer containing 20 mM Hepes, pH 7.4, 150 mM NaCl, and 0.1% digitonin. An initial screen assessing conformational homogeneity of hPANX1 in different detergents (C12E9, LMNG, and digitonin) identified digitonin as the top-performing reagent (Supplementary Fig. S1). Notably, we also found that protein concentration can significantly influence particle orientation in our cryo samples. Specifically, whereas low concentration cryo samples (3 mg/ml) showed a strong preference for top and bottom views (~95% of particles) (Supplementary Fig. S1d), high concentration cryo samples (8 mg/ml) preferred side view orientation, (~65% of particles) (Supplementary Fig. S1e). We therefore eluted the recombinant hPANX1 as a monodisperse peak under 0.1% digitonin followed by cryo-EM determination at a concentration of around 8 mg/ml. The peak corresponding to the hPANX1 channel was collected for further cryo-microscopy analysis.

### Cryo-electron microscopy

The cryo-EM grids were prepared using Vitrobot Mark IV (FEI) operated at 4 °C and 100% humidity. For samples of hPANX1 in digitonin, 4 μl aliquots of samples at concentrations of approximately 8 mg/ml were applied onto glow-discharged holey carbon grids (Quantifoil R1.2/1.3) 300 mesh Au grid. After a waiting time of 5 s, the grids were blotted for 2 s and plunged into liquid ethane for quick freezing.

The cryo-EM grids were screened on a Tecnai Arctica microscope (FEI) operated at 200 kV using a Falcon II 4k × 4k camera (FEI). The qualified grids were transferred into a Titan Krios microscope (FEI) operated at 300 kV for data acquisition equipped with Gatan K2 Summit detector and GIF Quantum energy filter. Images were automatically recorded using AutoEMation with a slit width of 20 eV on the energy filter and in the super-resolution mode at a nominal magnification of 130,000×, corresponding to a calibrated pixel size of 1.08 Å at the object scale, and with defocus ranging from 1.4 to 1.9 μm. Each stack was exposed for 5.6 s with an exposing time of 0.175 s per frame, resulting in a total of 32 frames per stack, and the total dose rate for each stack was about 50 e/Å^2^.

### Image processing

The flowchart for data processing was summarized in Supplementary Figs. S2 and S3. In total, 6241 micrographs (movie stacks) were collected for hPANX1. The global motion-corrected stacks (the first two frames were discarded) were further processed by sub-region motion correction and dose weighting using MotinCor2, generating summed micrographs with or without dose weighting. CTFFIND4 was used to estimate the contrast transfer function (CTF) parameters and produce the CTF power spectrum on the basis of summed micrographs from MotionCor2. Particles were auto-picked using Relion3.0^53^. For the dataset of hPANX1, ~1000 particles were manually picked in advance, and processed by 2D classification using Relion3.0^53^. The resulting 2D averages were served as the templates for particle picking. Two rounds of 2D classifications were performed to exclude noise and other bad particles. 1662k particles from qualified 2D averages were selected for further 3D analysis. Ahead of 3D classification, a round of refinement was applied on the whole particle sets using Relion3.0^53^. Three rounds of 3D classification with C1 symmetry generated 221k particles with good signal and round particle shape. Aiming at to get higher resolution, we proceeded substantial 3D classification with C7 symmetry and select 113k better particles. The particles were re-centered and processed by auto-refine with soft mask and C7 symmetry imposed using Relion3.0^53^. Finally, we obtained a 3.4 Å resolution map with C7 symmetry (Supplementary Fig. S2). The local resolution map was calculated using ResMap^54^, and displayed in Chimera^55^.

To achieve better resolution of the final map, the 221k particles from Relion were then transferred to the cryoSPARC^56^ software, followed by ab-initio reconstruction, heterogeneous refinement, non-uniform refinement, and local refinement, resulting in a 3.15 Å resolution map (Supplementary Fig. S3). The 3.15 Å map from cryoSPARC was almost identical to the 3.4 Å map from Relion, except that the CTD density from the 3.15 Å map was better than that of the 3.4 Å map.

To compensate regional resolution-dependent amplitude falloff, a LocScale map was produced by applying the Local Scaling Algorithm^34^ in a density window of 30 Å. This step facilitates visualization of the map as well as the later application of the mean-shift algorithm^35,36^ when the atomic model was constructed.

### Atomic modeling, refinement, and validation

The structural modeling of hPANX1-MD structure was completed in multiple stages. First, a preliminary model comprised mostly of conspicuous secondary structural motifs such as alpha-helix and beta-strands in NTH, TM1-4, E1H, and E2β1-3 was constructed using Phenix v1.16^57^ and COOT^58^, and we refer to this model as the raw hPANX1 model. At this stage, side-chains were fitted in-place only if they are supported by well-resolved densities; else, polyalanine sub-models were used as a place-holder for the structure. The raw hPANX1 model, consisting of 260 resolved side-chain residues and 88 unidentified alanine residues (mainly in CTH1-4 with discontinuity in loops), was inspected, where drafts for the unidentified side-chains were constructed in Modeller^59^ by sliding across the PANX1-Tag sequence. The resultant models were then sent for molecular dynamic flexible fitting (MDFF)^37,60^ with additional harmonic restraint on Cα. After collecting all the fitted drafts, they were inspected and selected by their global cross-correlation with the map. At this stage, the selected model still contains 86 missing residues mostly in concern with loops between CLH1 and CLH2 D162(162)-Y193(201) and in the C-terminal Q392(400)-C426(434). Atomic modeling there was impeded by regional resolution-dependent amplitude falloff that may attribute to conformational inhomogeneity in side-chains. To alleviate the falloff, a LocScale map^34^ was produced, where densities were strengthened in the inner layer of the channel where the heptameric C-termini meet up. The separation of chains in the C-termini at this stage was not entirely unambiguous in terms of isosurface. To trace the heptameric main chains there, we have implemented a version of mean-shift algorithm^36^ described in the MAINMAST method, which produces local dense points (LDPs) on relatively denser regions (Supplementary Fig. S12 and Movie S10). The mean-shift was applied to the LocScale map, where unequal distribution of density had been compensated. Subsequently, the main chain was extended to full-length manually by following closely on trails indicated by LDPs. A manual interactive adjustment was done using the AutoIMD package of VMD v1.9.3^61^. Next, the full-length model was sent for 18 rounds of Cascade MDFF (cMDFF)^38^; In between rounds, except for the last eight rounds, manual interactive adjustments were also done at the side-chain level to avoid undesirable minima. Finally, to establish strict C7 symmetry in the structure as well as to remove clashes and outliers in bond angles and dihedrals, phenix.real_space_refine (RSR) was employed to refine the model from the last cMDFF round. Successive rounds of RSR were conducted until Molprobity score^62^ converged.

Global fitness of models to the cryo-EM density was assessed by Fourier shell correlation (FSC) curves; correlation was made between the experimental map and voxels produced by assigning Gaussian blur of width 3.15 Å to each atomic coordinate. The global assessment was performed for both masked and unmasked maps. In successive rounds of cMDFF, atom-to-map resolution determined at FSC = 0.5 had improved from 4.96 to 3.74 Å (Supplementary Fig. S12a, b). The hPANX1-MD model produced by applying RSR on the final cMDFF model further improves the resolution to 3.5 Å (Supplementary Fig. S12a). In addition, we also assessed the local fitness of our models through the SMOC *Z*-score^63,64^ of the TEMPy package^65^, where *Z*-scoring was done by collecting standard deviation and average of the SMOC score across all residues and all models (Supplementary Fig. S12c, d). During the course of cMDFF, local correlation improves gradually. In particular, the effect of RSR is obvious in CLH1 residues 100(100)-160(160), TM3 residue 202(210)-242(250), and CTH1 residues 293(301)-311(319). Surface charges were calculated with Adaptive Poisson–Boltzmann Solver 1.3^66^.

### Molecular dynamics (MD) simulations

Our MD simulations were done in NAMD v2.1.2^67^ with the CHARMM36 all-atom forcefield^68^. The Particle Mesh Ewald (PME) method^69^ was applied on electrostatics; periodic boundary was in place. All systems were propagated in 1.5 fs time-steps. Temperature and pressure were maintained at 310 K and 1 atm by Langevin dynamics. Simulations were conducted on a model of PANX1 wildtype sequence instead of the experimental hPANX1-MD model as the inserted Strep-tag residue (173–181, WSHPQFEK) can occlude the early intracellular opening of the channel (Fig. 4a). To establish the PANX1 model, residues (173–181) were removed from the experimental hPANX1-MD structure and residue 172(172) was then joined to residue 173(182). The system, in vacuum, was then minimized under harmonic restraint on all atoms except for those of residue 162(162)–184(192), i.e., ±10 residues from the deletion. As in the hPANX1-MD model, Met1 was absent in all simulations. Sidechains were protonated according to standard neutral conditions; histidines were always protonated in position *δ*. Disulfide bonds identified by distance in the experimental hPANX1-MD structure were maintained in the PANX1 model.

To initialize the simulation, the PANX1 protein was first solvated with explicit TIP3P water using the Solvate v.1.0.1 plugin of VMD and then a water layer of 3 nm thick in the *z*-axis was removed, with an exception for the cylinder in the central channel, to assure that the transmembrane regions were exposed for lipid embedding. The transmembrane regions were chosen by considering surface charge distribution (Supplementary Fig. S15d) and were inspected to overlap with the amphipol shell observed in the experimental cryo-EM data. Using the Membrane-Builder plugin of VMD, a 1-palmitoyl-2-oleoyl-sn-glycerol-3-phosphocholine (POPC) lipid bilayer of dimension 127 Å * 127 Å was inserted; lipids overlapping with the protein were removed. To assure the absence of gaps between the inserted lipid and water, the entire system was solvated once again. Finally, the system was neutralized using the Autoionize v1.5 plugin of VMD with salt concentration set at 0.8 M for KCl and 0.8 M for CaCl_2_; the relatively high salt concentration was chosen in agreement with studies conducted on sodium and potassium channels^70–73^. After building up the system, to avoid clashes in solvent and lipid constructs, a preliminary energy minimization, under harmonic restraint on heavy atoms of the protein and hydrophilic heads of the lipid, was performed for 1.5 ns. Subsequently, harmonic restraints on the lipid were also released and the whole system was allowed to propagate in random velocities to “melt” the lipid bilayer for 4.5 ns. The “molten” configuration of the system was illustrated in Supplementary Fig. S15d. Next, as an equilibration run, the entire system—except for backbone heavy atoms on TM1–4—was free from restraints to propagate for 25 ns (Supplementary Fig. S15c, gray background). Finally, in the production run, all harmonic restraints were released and the system was allowed to propagate for an additional 100 ns (Supplementary Fig. S15c, white background). Root mean squared deviations (r.m.s.d.) and root mean squared fluctuations (r.m.s.f.) against *C*_α_ were monitored during equilibration and production runs (Supplementary Fig. S15a–c). Cα of the experimental structure (with Strep-tag excluded) was used as a reference for the calculation. In particular, r.m.s.d. for the N-termini/C-termini and the transmembrane helices were also examined. All domains, as well as the overall structure, approached a steady r.m.s.d. within runtime (Supplementary Fig. S15c).

### Umbrella sampling and ion sampling profile

In this study, three ion permeation profile calculations (calcium, chloride, and potassium) were set up. Of each, the weighted histogram analysis method (WHAM)^74^ was used to estimate the potential of mean force (PMF) associated with the ion permeation process, where the displacement along the *z*-axis, which is normal to the lipid bilayer, was chosen as the reaction coordinate *q*. The subject of WHAM analysis is a set of umbrella sampling (US) trajectories. Prior to US simulations, inspection of the production run MD trajectory reveals that, even though the channel is not dewetted, ions cannot get past major constrictions at E414 and S424 within a period of 100 ns (Supplementary Fig. S15e–g). Therefore, to assure sufficient sampling, 20 rounds of steered MD (SMD), which each pulls one designated ion near N412 in *z*-direction, was applied to seed the channel from +5.5 to −5.5 nm at an interval of 0.1 nm. Subsequently, five snapshots of the SMD trajectories were chosen at each interval to initiate independent US simulations. To summarize, 5 * 110 independent US simulations each spanning 9 ns was obtained. In each US trajectory, the ion seeded at unique position *q*_i_ was constrained by a biasing harmonic potential *U*_i_ to assure effective sampling of coordinate *q*, where *c* is a uniform constant at 20 kcal/mol/Å^2^ used to control the strength of bias.

$$U_{\rm{i}} = c/2\left( {q - q_{\rm{i}}} \right)^2$$

After completing all the US simulations, WHAM was then performed by mapping *q* of the trajectories into bins of width 0.05 nm. We index each trajectory and its *U* by subscript *i* ∈ {0, 1, …, *R*} and *j* ∈ {0, 1, …, *R*}; the total number of data points taken from the *i*th trajectory is *n*_i_. Each windowing bin in the histogram is indexed by *k* ∈ {0, 1, …, *L*}; count of data point at the *k*th bin is *N*_k_. *β* is the product of Boltzmann constant *k*_B_ (1.38064852 × 10^–23^ m^2^ kg s^−2^ K^−1^) and a uniform temperature (310 K). The unbiased probability *P* and the PMF of the system *A* can then be obtained by minimizing the statistical error as outlined in ref. ^74^.

$$\begin{array}{l}P\left( q \right) = \mathop {\sum}\limits_{k = 1}^L {N_k \ast {\mathrm{exp}}\left( { - \beta \mathop {\sum}\limits_{j = 0}^R {\lambda _{\rm{j}}U_{\rm{j}}\left( {q,q_{\rm{j}}} \right)} } \right)} /\mathop {\sum}\limits_{i = 1}^R {n_i} \\ \ast {\mathrm{exp}}\left( {\beta A_{\rm{i}} - \beta \mathop {\sum}\limits_{J = 0}^R {\lambda _{\rm{j}}U_{\rm{j}}\left( {q,q_{\rm{j}}} \right)} } \right) \\ A\left( q \right) = - \beta ^{ - 1}\,{\mathrm{lnP}}\left( q \right)\end{array}$$

In practice, both *P* and *A* are unknowns and they need to be determined by iteration under normalization constraint on the denominator of *P* (i.e., the weights). In our case, an efficient solver from PyEMMA2^75^ was used in this regard. To satisfy differentiability, the obtained discrete PMF was smoothened by fitting quadratic functions. In Extended Data Fig. 8i, we show convergence of our PMF profiles over 8 ns. Each ion permeation profile requires 106 core hours of computation.

### Statistical analysis

Data are presented as the mean ± standard deviation (s.d.) or mean ± standard error of the mean (s.e.m.). Unpaired two-tailed Student’s *t*-test or one-way ANOVA with Dunnett’s test were used for *P* value calculation, unless stated otherwise. A *P* value less than 0.05 was considered statistically significant. The 95% confidence intervals for comparison of Cα rmsf values were calculated using a two-tailed Student’s *t*-test. For cross-validation against overfitting^76^, we randomly displaced the atom positions of the final model by up to a maximum of 0.5 Å, and refined against the half map 1 generated by RELION 3D auto-refine procedure, resulting in a model named test. Then we calculated the FSC curve of both half map against the model test, and compared with the FSC curve of final model against the summed map generated by Relion 3D auto-refine procedure.

### In-gel pepsin digestion

Gel bands of proteins were excised for in-gel digestion, and proteins were identified by mass spectrometry. Briefly, the gel bands were reduced with 25 mM of dithiothreitol (DTT) and alkylated with 55 mM Iodoacetamide. In-gel digestion was performed using Pepsin (Promega) in 1% formic acid at 37 °C overnight. The peptides were extracted twice with 1% trifluoroacetic acid in 50% acetonitrile aqueous solution for 30 min. The peptide extracts were then centrifuged in a SpeedVac to reduce the volume.

### LC-MS/MS analysis

The digestion products were separated by a 60 min gradient elution at a flow rate of 0.300 µL/min with the EASY-nLC 1000 system which was directly interfaced with the Thermo Orbitrap Fusion mass spectrometer. The analytical column was purchased from Thermofisher (50 μm ID, 150 mm length; packed with C-18 resin). Mobile phase A consisted of 0.1% formic acid, and mobile phase B consisted of 100% acetonitrile and 0.1% formic acid. The Orbitrap Fusion mass spectrometer was operated in the data-dependent acquisition mode using Xcalibur3.0 software and there is a single full-scan mass spectrum in the Orbitrap (350–1550 *m/z*, 120,000 resolution) followed by top-speed MS/MS scans in the Orbitrap. The MS/MS spectra from each LC-MS/MS run were searched against the selected database using Byonic and Biologic software (Protein Metrics, CA).

## Supplementary information

raw data for gels and blots

Supplementary information

editorial policy checklist

reporting summay

Supplementary Video 1

Supplementary Video 2

Supplementary Video 3

Supplementary Video 4

Supplementary Video 5

Supplementary Video 6

Supplementary Video 7

Supplementary Video 8

Supplementary Video 9

Supplementary Video 10

## Data Availability

The 3D cryo-electron microscopy density raw map, local scale sharpened map and phenix sharpened map have been deposited in the Electron Microscopy Data Bank (EMDB), with accession code EMD-30880, EMD-30881, and EMD-31025, respectively. The coordinates of PANX1-MD atomic model have been deposited in the Protein Data Bank (PDB), with the accession code 7DWB.

## References

[1] Bruzzone R, Hormuzdi SG, Barbe MT, Herb A, Monyer H (2003). Pannexins, a family of gap junction proteins expressed in brain. Proc Natl Acad. Sci. USA.

[2] Penuela S, Gehi R, Laird DW (2013). The biochemistry and function of pannexin channels. Biochim Biophys Acta.

[3] Silverman WR (2009). The Pannexin 1 channel activates the inflammasome in neurons and astrocytes. J. Biol. Chem..

[4] Wang JJ (2014). The membrane protein Pannexin1 forms two open-channel conformations depending on the mode of activation. Sci. Signal..

[5] Chekeni FB (2010). Pannexin 1 channels mediate ‘find-me’ signal release and membrane permeability during apoptosis. Nature.

[6] Locovei S, Wang JJ, Dahl G (2006). Activation of pannexin 1 channels by ATP through P2Y receptors and by cytoplasmic calcium. FEBS Lett..

[7] Bao L, Locovei S, Dahl G (2004). Pannexin membrane channels are mechanosensitive conduits for ATP. FEBS Lett..

[8] Sridharan M (2010). Pannexin 1 is the conduit for low oxygen tension-induced ATP release from human erythrocytes. Am. J. Physiol. Heart Circ. Physiol..

[9] Chiu YH, Schappe MS, Desai BN, Bayliss DA (2018). Revisiting multimodal activation and channel properties of Pannexin 1. J. Gen. Physiol..

[10] Dahl, G. The Pannexin1 membrane channel: distinct conformations and functions. *FEBS Lett*. **592**, 3201–3209 (2018). 10.1002/1873-3468.13115 29802622

[11] Chiu YH (2017). A quantized mechanism for activation of pannexin channels. Nat. Commun..

[12] Sandilos JK (2012). Pannexin 1, an ATP release channel, is activated by caspase cleavage of its pore-associated C-terminal autoinhibitory region. J. Biol. Chem..

[13] Dourado M, Wong E, Hackos DH (2014). Pannexin-1 is blocked by its C-terminus through a delocalized non-specific interaction surface. PLoS ONE.

[14] Michalski K (2020). The cryo-EM structure of a pannexin 1 reveals unique motifs for ion selection and inhibition. Elife.

[15] Qu RG (2020). Cryo-EM structure of human heptameric Pannexin 1 channel. Cell Res..

[16] Mou L (2020). Structural basis for gating mechanism of Pannexin 1 channel. Cell Res..

[17] Jin Q (2020). Cryo-EM structures of human pannexin 1 channel. Cell Res..

[18] Deng Z (2020). Cryo-EM structures of the ATP release channel pannexin 1. Nat. Struct. Mol. Biol..

[19] Ruan Z, Orozco IJ, Du J, Lu W (2020). Structures of human pannexin 1 reveal ion pathways and mechanism of gating. Nature.

[20] Wang JJ, Dahl G (2010). SCAM analysis of Panx1 suggests a peculiar pore structure. J. Gen. Physiol..

[21] Yang D, He Y, Munoz-Planillo R, Liu Q, Nunez G (2015). Caspase-11 requires the Pannexin-1 channel and the purinergic P2X7 pore to mediate pyroptosis and endotoxic shock. Immunity.

[22] Boassa D (2007). Pannexin1 channels contain a glycosylation site that targets the hexamer to the plasma membrane. J. Biol. Chem..

[23] Penuela S, Bhalla R, Nag K, Laird DW (2009). Glycosylation regulates pannexin intermixing and cellular localization. Mol. Biol. Cell.

[24] Pelegrin P, Surprenant A (2006). Pannexin-1 mediates large pore formation and interleukin-1 beta release by the ATP-gated P2X(7) receptor. EMBO J..

[25] Wells CA (2013). Stemformatics: visualisation and sharing of stem cell gene expression. Stem Cell Res..

[26] Choi J (2019). Stemformatics: visualize and download curated stem cell data. Nucleic Acids Res..

[27] Xue J (2014). Transcriptome-based network analysis reveals a spectrum model of human macrophage activation. Immunity.

[28] Reynier F (2012). Gene expression profiles in alveolar macrophages induced by lipopolysaccharide in humans. Mol. Med..

[29] Tailleux L (2008). Probing host pathogen cross-talk by transcriptional profiling of both Mycobacterium tuberculosis and infected human dendritic cells and macrophages. PLoS ONE.

[30] Zhang HR (2015). Functional analysis and transcriptomic profiling of ipsc-derived macrophages and their application in modeling mendelian disease. Circ. Res..

[31] Douanne T (2019). Pannexin-1 limits the production of proinflammatory cytokines during necroptosis. EMBO Rep..

[32] Bergsbaken T, Fink SL, Cookson BT (2009). Pyroptosis: host cell death and inflammation. Nat. Rev. Microbiol..

[33] Maeda S (2009). Structure of the connexin 26 gap junction channel at 3.5 angstrom resolution. Nature.

[34] Jakobi AJ, Wilmanns M, Sachse C (2017). Model-based local density sharpening of cryo-EM maps. Elife.

[35] Fukunaga K, Hostetler LD (1975). Estimation of gradient of a density-function, with applications in pattern-recognition. IEEE Trans. Inform. Theory.

[36] Terashi G, Kihara D (2018). De novo main-chain modeling for EM maps using MAINMAST. Nat. Commun..

[37] Trabuco LG, Villa E, Mitra K, Frank J, Schulten K (2008). Flexible fitting of atomic structures into electron microscopy maps using molecular dynamics. Structure.

[38] Singharoy A (2016). Molecular dynamics-based model refinement and validation for sub-5 angstrom cryo-electron microscopy maps. Elife.

[39] Qiu F, Dahl G (2009). A permeant regulating its permeation pore: inhibition of pannexin 1 channels by ATP. Am. J. Physiol. Cell Physiol..

[40] Guthrie PB (1999). ATP released from astrocytes mediates glial calcium waves. J. Neurosci..

[41] Ma WH (2012). Pannexin 1 forms an anion-selective channel. Pflug. Arch..

[42] Qu Y (2011). Pannexin-1 is required for ATP release during apoptosis but not for inflammasome activation. J. Immunol..

[43] Shi JJ (2015). Cleavage of GSDMD by inflammatory caspases determines pyroptotic cell death. Nature.

[44] Jackson DG, Wang JJ, Keane RW, Scemes E, Dahl G (2014). ATP and potassium ions: a deadly combination for astrocytes. Sci. Rep..

[45] Suadicani SO (2012). ATP signaling is deficient in cultured pannexin1-null mouse astrocytes. Glia.

[46] Munoz-Planillo R (2013). K+ efflux is the common trigger of NLRP3 inflammasome activation by bacterial toxins and particulate matter. Immunity.

[47] Li S (2011). Expression and roles of pannexins in ATP release in the pituitary gland. Endocrinology.

[48] Kranz K (2013). Expression of Pannexin1 in the outer plexiform layer of the mouse retina and physiological impact of its knockout. J. Comp. Neurol..

[49] Cowan KN, Langlois S, Penuela S, Cowan BJ, Laird DW (2012). Pannexin1 and Pannexin3 exhibit distinct localization patterns in human skin appendages and are regulated during keratinocyte differentiation and carcinogenesis. Cell Commun. Adhes..

[50] Chen W (2019). Enhanced macrophage pannexin 1 expression and hemichannel activation exacerbates lethal experimental sepsis. Sci. Rep..

[51] Locovei S, Scemes E, Qiu F, Spray DC, Dahl G (2007). Pannexin1 is part of the pore forming unit of the P2X(7) receptor death complex. FEBS Lett..

[52] Wicki-Stordeur LE, Swayne LA (2014). The emerging Pannexin 1 signalome: a new nexus revealed?. Front. Cell Neurosci..

[53] Zivanov J (2018). New tools for automated high-resolution cryo-EM structure determination in RELION-3. Elife.

[54] Kucukelbir A, Sigworth FJ, Tagare HD (2014). Quantifying the local resolution of cryo-EM density maps. Nat. Methods.

[55] Pettersen EF (2004). UCSF Chimera-a visualization system for exploratory research and analysis. J. Comput. Chem..

[56] Punjani A, Rubinstein JL, Fleet DJ, Brubaker MA (2017). cryoSPARC: algorithms for rapid unsupervised cryo-EM structure determination. Nat. Methods.

[57] Adams PD (2010). PHENIX: a comprehensive Python-based system for macromolecular structure solution. Acta Crystallogr. D. Biol. Crystallogr..

[58] Emsley P, Cowtan K (2004). Coot: model-building tools for molecular graphics. Acta Crystallogr. D. Biol. Crystallogr..

[59] Fiser A, Do RK, Sali A (2000). Modeling of loops in protein structures. Protein Sci..

[60] Chan KY (2011). Symmetry-restrained flexible fitting for symmetric EM maps. Structure.

[61] Humphrey W, Dalke A, Schulten K (1996). VMD: Visual molecular dynamics. J. Mol. Graph. Model.

[62] Davis IW (2007). MolProbity: all-atom contacts and structure validation for proteins and nucleic acids. Nucleic Acids Res..

[63] Joseph AP (2016). Refinement of atomic models in high resolution EM reconstructions using Flex-EM and local assessment. Methods.

[64] Joseph AP, Lagerstedt I, Patwardhan A, Topf M, Winn M (2017). Improved metrics for comparing structures of macromolecular assemblies determined by 3D electron-microscopy. J. Struct. Biol..

[65] Farabella I (2015). TEMPy: a Python library for assessment of three-dimensional electron microscopy density fits. J. Appl. Crystallogr..

[66] Baker NA, Sept D, Joseph S, Holst MJ, McCammon JA (2001). Electrostatics of nanosystems: application to microtubules and the ribosome. Proc. Natl Acad. Sci. USA.

[67] Phillips JC (2005). Scalable molecular dynamics with NAMD. J. Comput. Chem..

[68] Huang J, MacKerell AD (2013). CHARMM36 all-atom additive protein force field: Validation based on comparison to NMR data. J. Comput. Chem..

[69] Darden T, York D, Pedersen L (1993). Particle mesh Ewald—an N.Log(N) method for Ewald sums in large systems. J. Chem. Phys..

[70] Berneche S, Roux B (2003). A microscopic view of ion conduction through the K+ channel. Proc. Natl Acad. Sci. USA.

[71] Corry B, Thomas M (2012). Mechanism of ion permeation and selectivity in a voltage gated sodium channel. J. Am. Chem. Soc..

[72] Ulmschneider MB (2013). Molecular dynamics of ion transport through the open conformation of a bacterial voltage-gated sodium channel. Proc. Natl Acad. Sci. USA.

[73] Kopfer DA (2014). Ion permeation in K+ channels occurs by direct Coulomb knock-on. Science.

[74] Kumar S, Bouzida D, Swendsen RH, Kollman PA, Rosenberg JM (1992). The weighted histogram analysis method for free-energy calculations on biomolecules. 1. The Method. J. Comput. Chem..

[75] Scherer MK (2015). PyEMMA 2: a software package for estimation, validation, and analysis of markov models. J. Chem. Theory Comput..

[76] Amunts A (2014). Structure of the yeast mitochondrial large ribosomal subunit. Science.

