# Supplementary figures and images for: Structure of the full-length human Pannexin1 channel and insights into its role in pyroptosis
Source: Cell Discov. 2021 May 4;7:30. doi: 10.1038/s41421-021-00259-0 (PMC8096850; doi:10.1038/s41421-021-00259-0)

Supplementary Fig. S11c

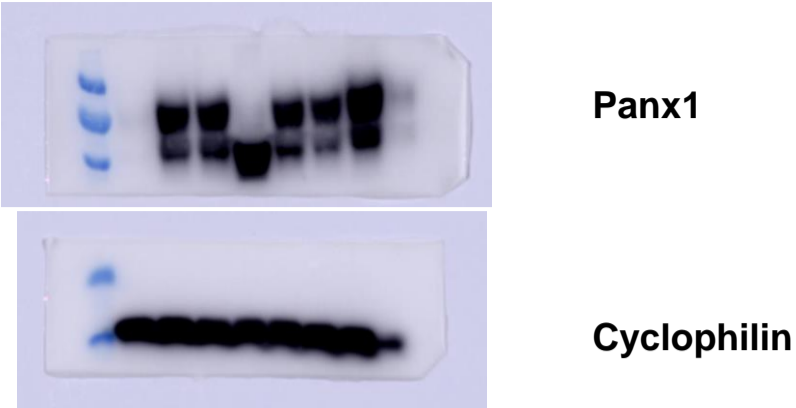

Supplementary Fig. S9b

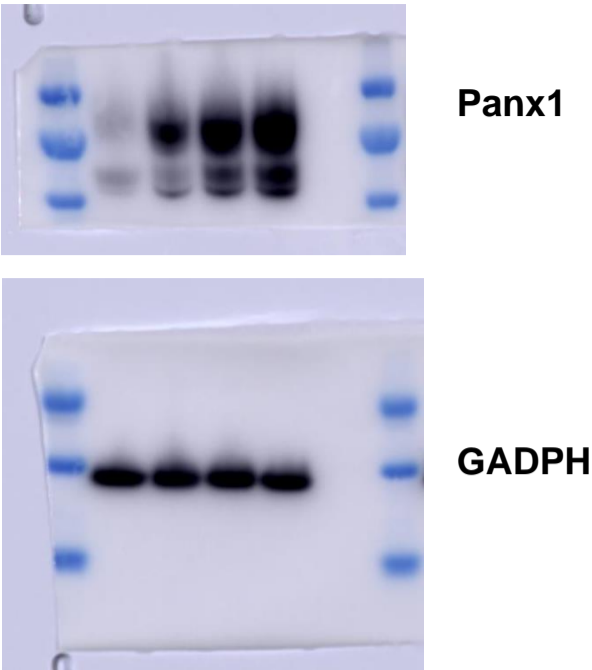

Figure 2c and 4b

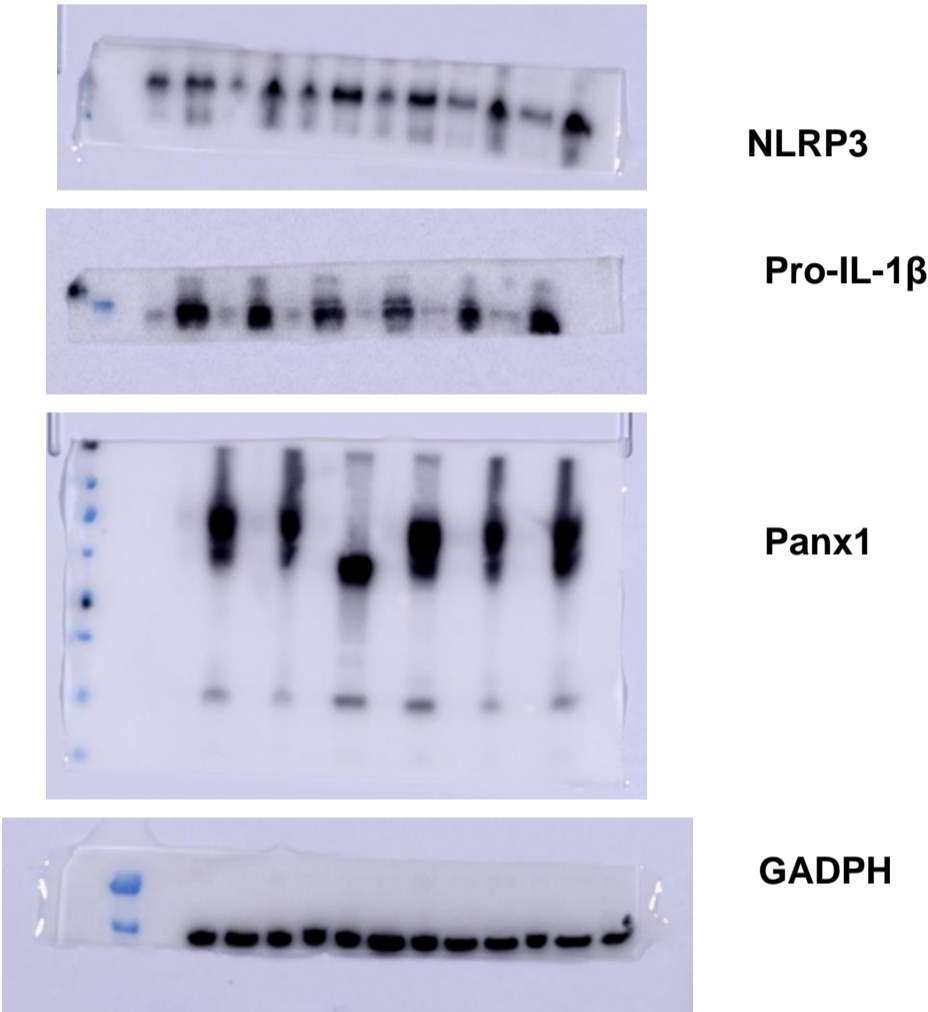

Supplementary Fig. S1a,b

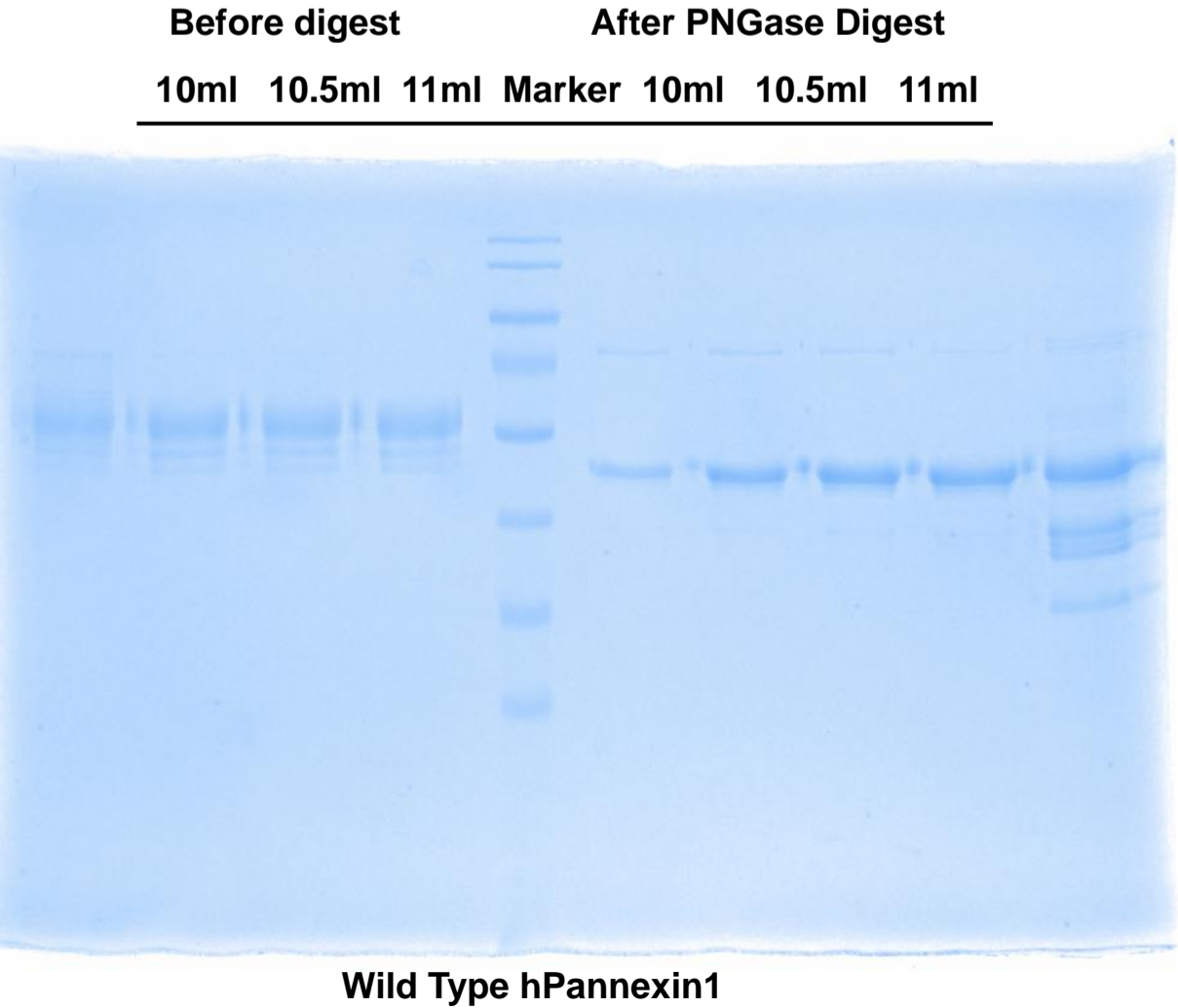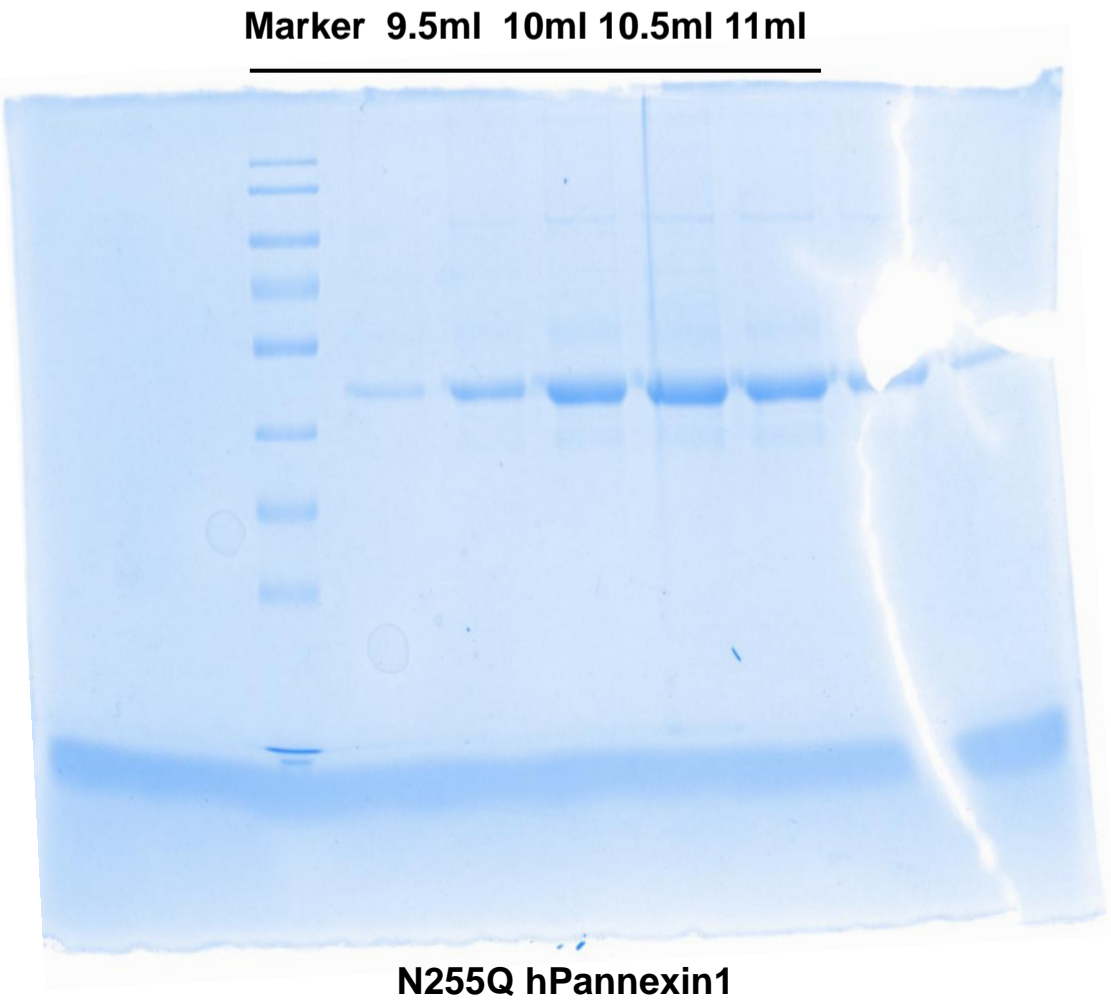

Supplement: Supplementary file 1 — raw data for gels and blots [file 41421_2021_259_MOESM1_ESM.pdf]
