# Supplementary material for: Structure of the full-length human Pannexin1 channel and insights into its role in pyroptosis
Source: Cell Discov. 2021 May 4;7:30. doi: 10.1038/s41421-021-00259-0 (PMC8096850; doi:10.1038/s41421-021-00259-0)
Supplement: Supplementary file 2 — Supplementary information [file 41421_2021_259_MOESM2_ESM.pdf]

Supplementary Materials for

Structure of the full-length human Pannexin1 channel and insights into its role in pyroptosis

Sensen Zhang[1,*], Baolei Yuan[2,*], Jordy Homing Lam[3,4*], Jun Zhou[1], Xuan Zhou[2], Gerardo Ramos-Mandujano[2], Xueyuan Tian[5], Yang Liu[1], Renmin Han[3], Yu Li[3], Xin Gao[3,#], Mo Li[2,#], Maojun Yang[1,6,#]

[1]Ministry of Education Key Laboratory of Protein Science, Tsinghua-Peking Center for Life Sciences, Beijing Advanced Innovation Center for Structural Biology, Beijing Frontier Research Center for Biological Structure, School of Life Sciences, Tsinghua University, Beijing 100084, China
[2]Laboratory of Stem Cell and Regeneration, Biological and Environmental Sciences and Engineering (BESE) Division, King Abdullah University of Science and Technology (KAUST), Thuwal 23955-6900, Kingdom of Saudi Arabia
[3]Computational Bioscience Research Center (CBRC), Computer, Electrical and Mathematical Sciences and Engineering (CEMSE) Division, King Abdullah University of Science and Technology (KAUST), Thuwal 23955-6900, Kingdom of Saudi Arabia
[4]Currentt address: Bridge Institute, USC Michelson Center for Convergent Biosciences, University of Southern California, Los Angeles, CA, USA.
[5]Peking-Tsinghua Center for Life Sciences, Academy for Advanced Interdisciplinary Studies, Peking University, Beijing, 100871, China
[6]School of Pharmacy, Tongji Medical College, Huazhong University of Science and Technology, Wuhan, 430030, China

[*]These authors contributed equally to this work.

[#]Correspondence: Xin Gao: xin.gao@kaust.edu.sa; Mo Li: mo.li@kaust.edu.sa; Maojun Yang: maojunyang@tsinghua.edu.cn

This file includes:

Supplementary Figs. S1 to S15

Supplementary Movie S1 to S10

Supplementary Table S1

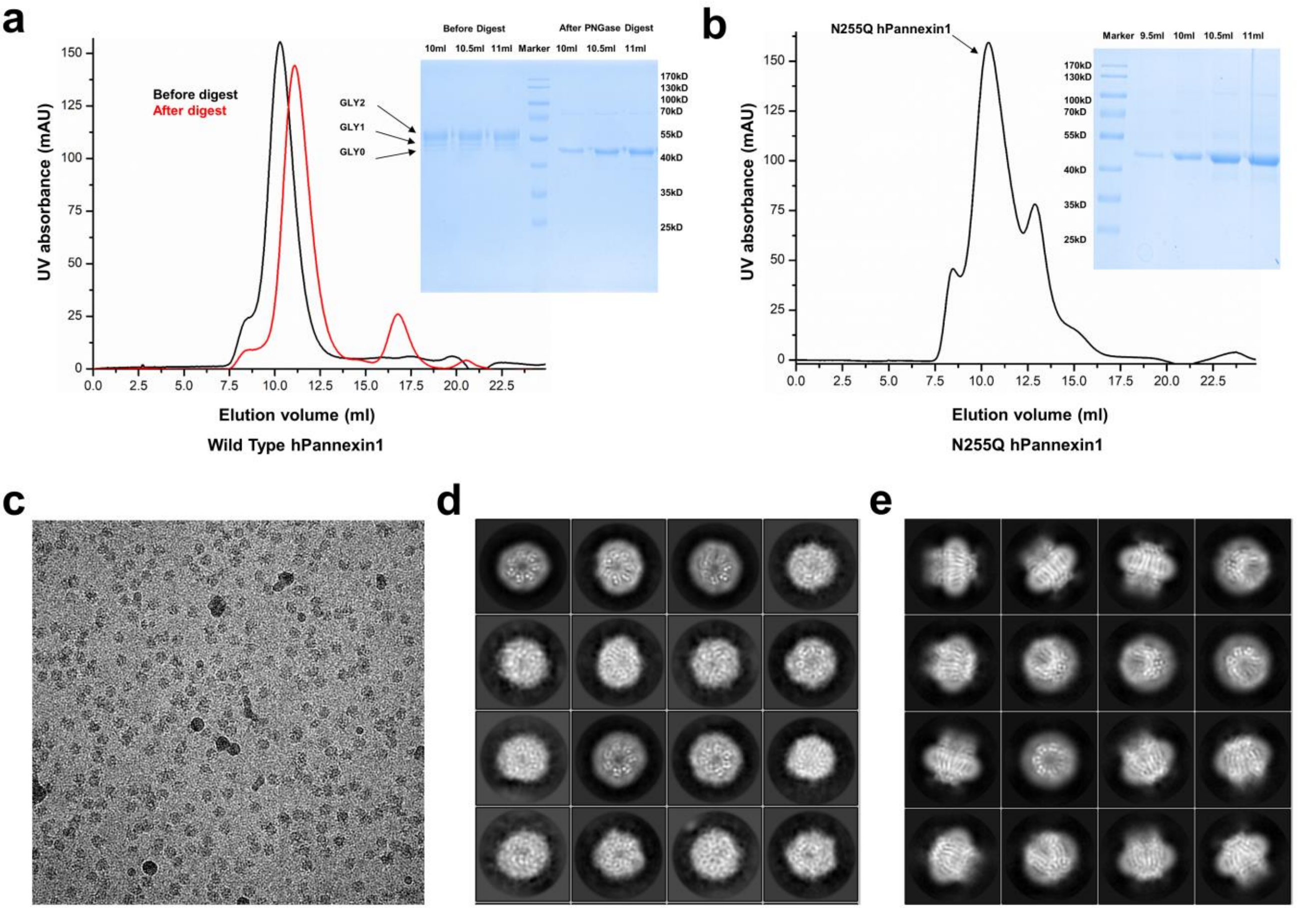

Supplementary Fig. S1. Purification and Cryo-EM study of hPANX1.

a. Size-exclusion chromatography of the wild type hPANX1 in the digitonin buffer. The peaks corresponding to the hPANX1 heptamer (before digest and after PNGase digest) are indicated. The corresponding peak from gel-filtration migrates as three bands (GLY2, GLY1 and GLY0) on SDS-PAGE and only exhibits one band (GLY0) after PNGase F glycosidase digestion.

b. Size-exclusion chromatography of N255Q hPANX1 in the digitonin buffer.

c. Representative cryo-EM micrograph of hPANX1 in digitonin.

d. 2D class averages of hPANX1 sample with a concentration of 3mg/ml in the digitonin buffer. Samples show a strong orientation preference as nearly 95% of the particles exhibit top and bottom views

e. 2D class averages of the hPANX1 sample with a concentration of 8 mg/ml in the digitonin buffer.

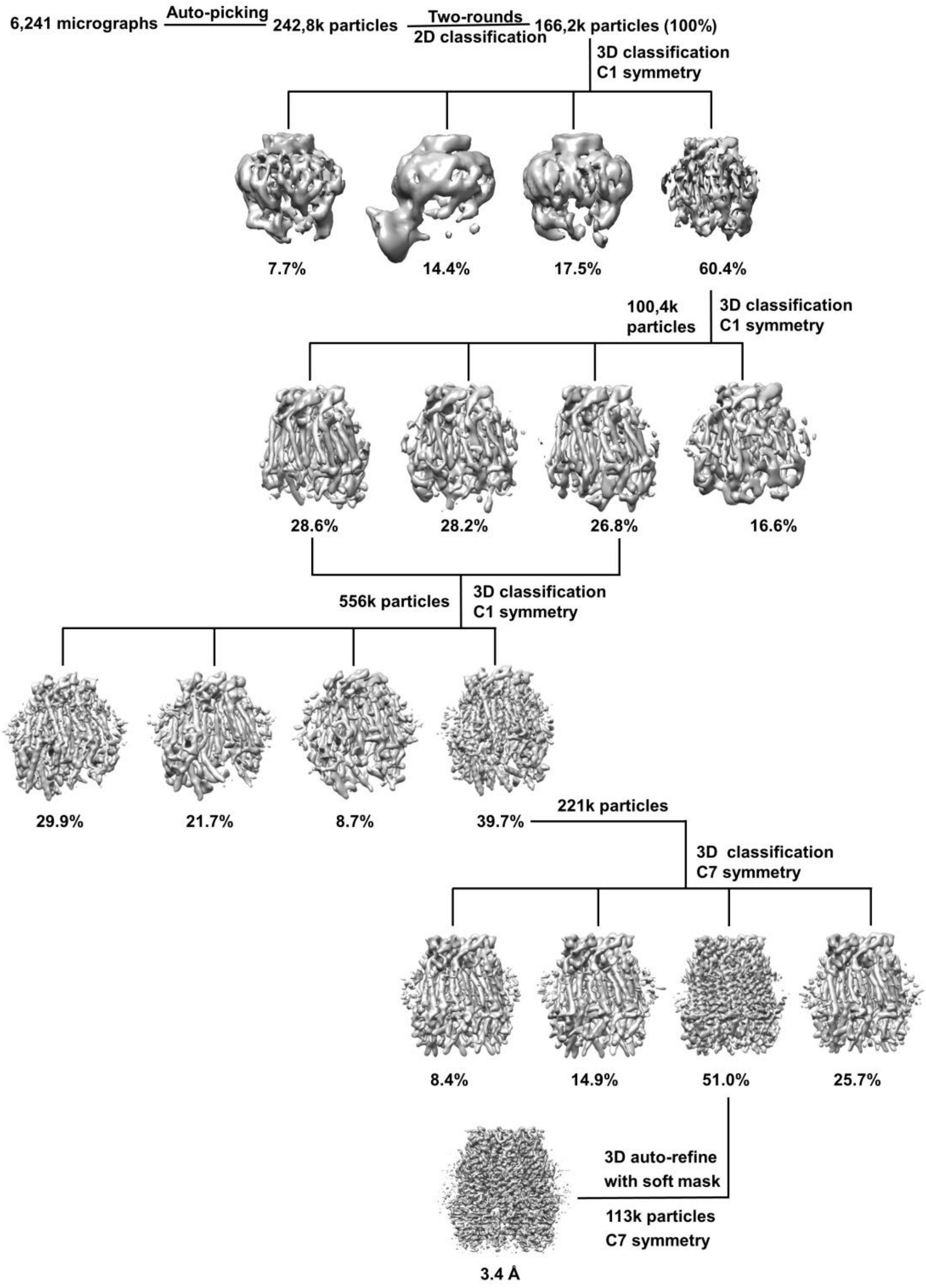

Supplementary Fig. S2. Workflow of data processing of hPANX1 in RELION.

Workflow of 2D/3D reconstruction with Cryo-EM data in RELION software. In brief, 166,2k particles were kept after 2D classification, and subject to two rounds of 3D classification. A final dataset containing 113k particles was used for high-resolution refinement (see methods for more details).

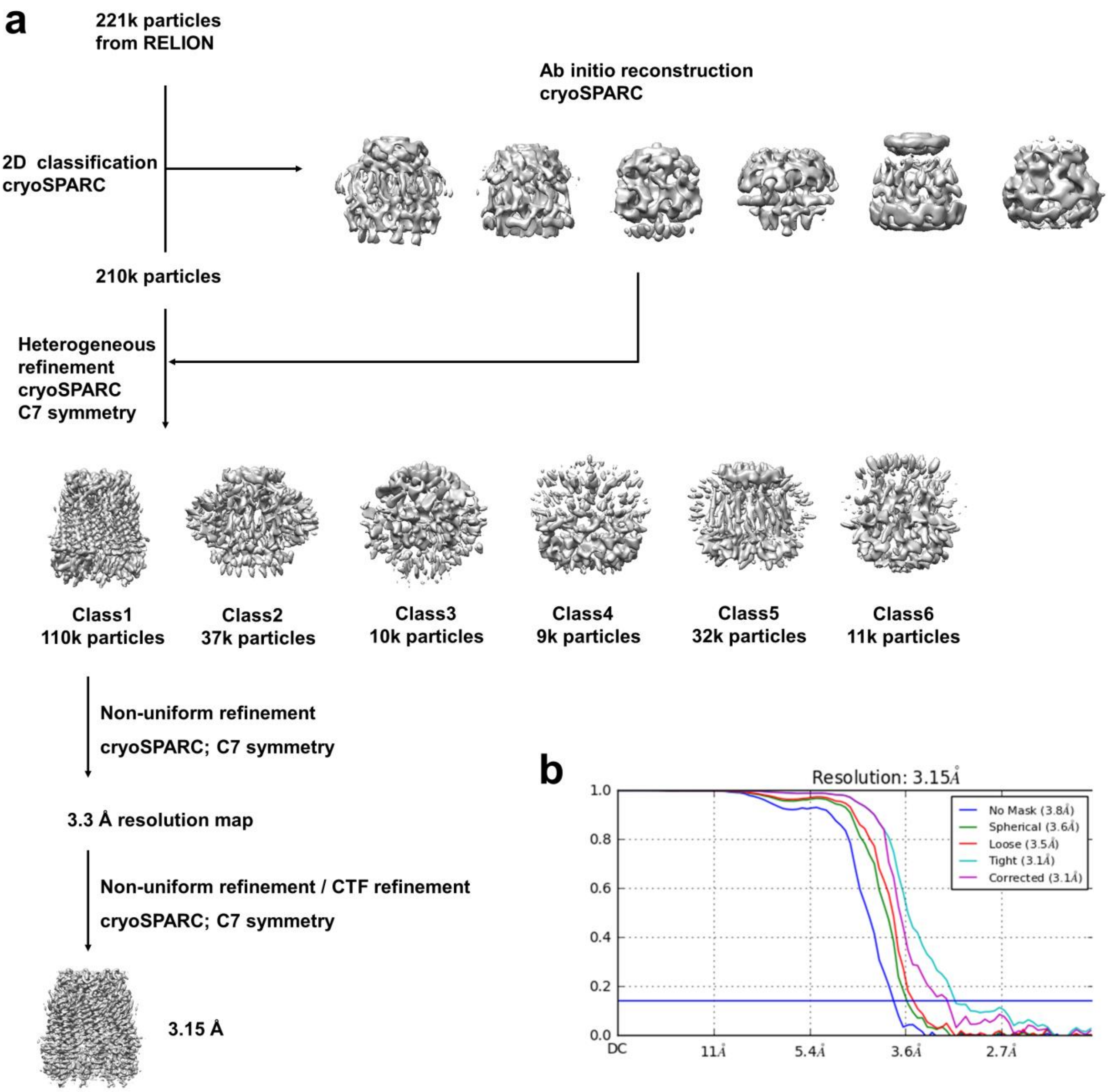

Supplementary Fig. S3. Workflow of data processing of hPANX1 in cryoSPARC.

a. Workflow of 3D reconstruction of hPANX1 with cryoSPARC software. In brief, 221k particles were served as input from the RELION results, and subject to heterogeneous refinement and non-unifor refinement. A final dataset containing 110k particles was used for high-resolution refinement after CTF refinement (see methods for more details).
b. Fourier Shell Correlation (FSC) cureves of hPANX1 after 3D refinement in cryoSPAC software. The cyan cures indicate the high-resolution map with an automatic tight mask (noise-substitution corrected).

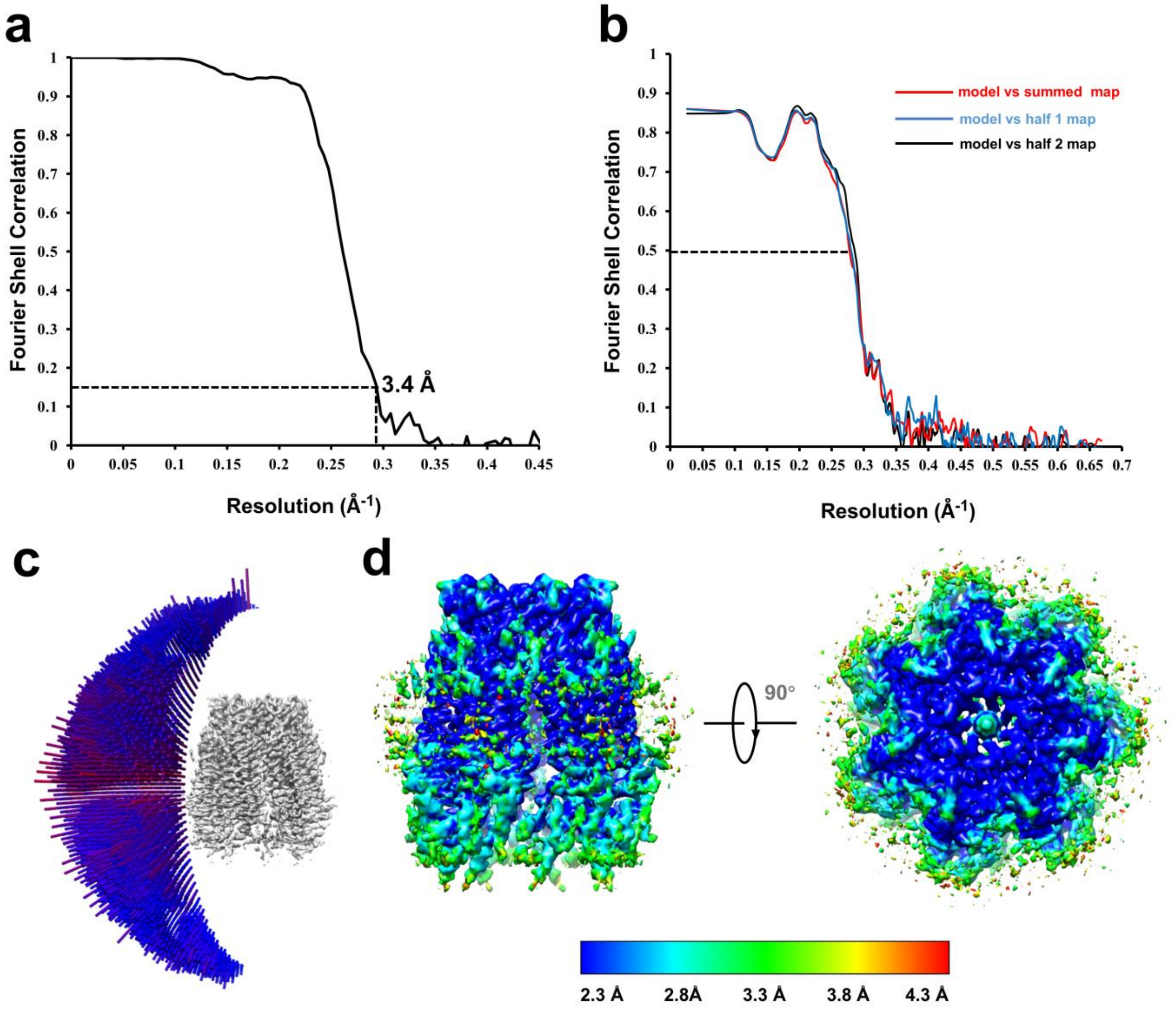

Supplementary Fig. S4. Structural determination of hPANX1.

a. Gold-standard Fourier Shell correlation (FSC) curves of hPANX1 after 3D refinement. Resolution estimation of hPANX1 (3.4 Å, red) from Relion was based on the criterion of FSC 0.143 cutoff.

b. Cross-validation of the atomic model with the summed map and the half maps of the raw hPANX1 model based on FSC = 0.5 curves.

c. Angular distribution of the raw hPANX1 map final reconstruction.

d. Local resolution map of the raw hPANX1 final 3D density map. From left to right are side and top views, respectively.

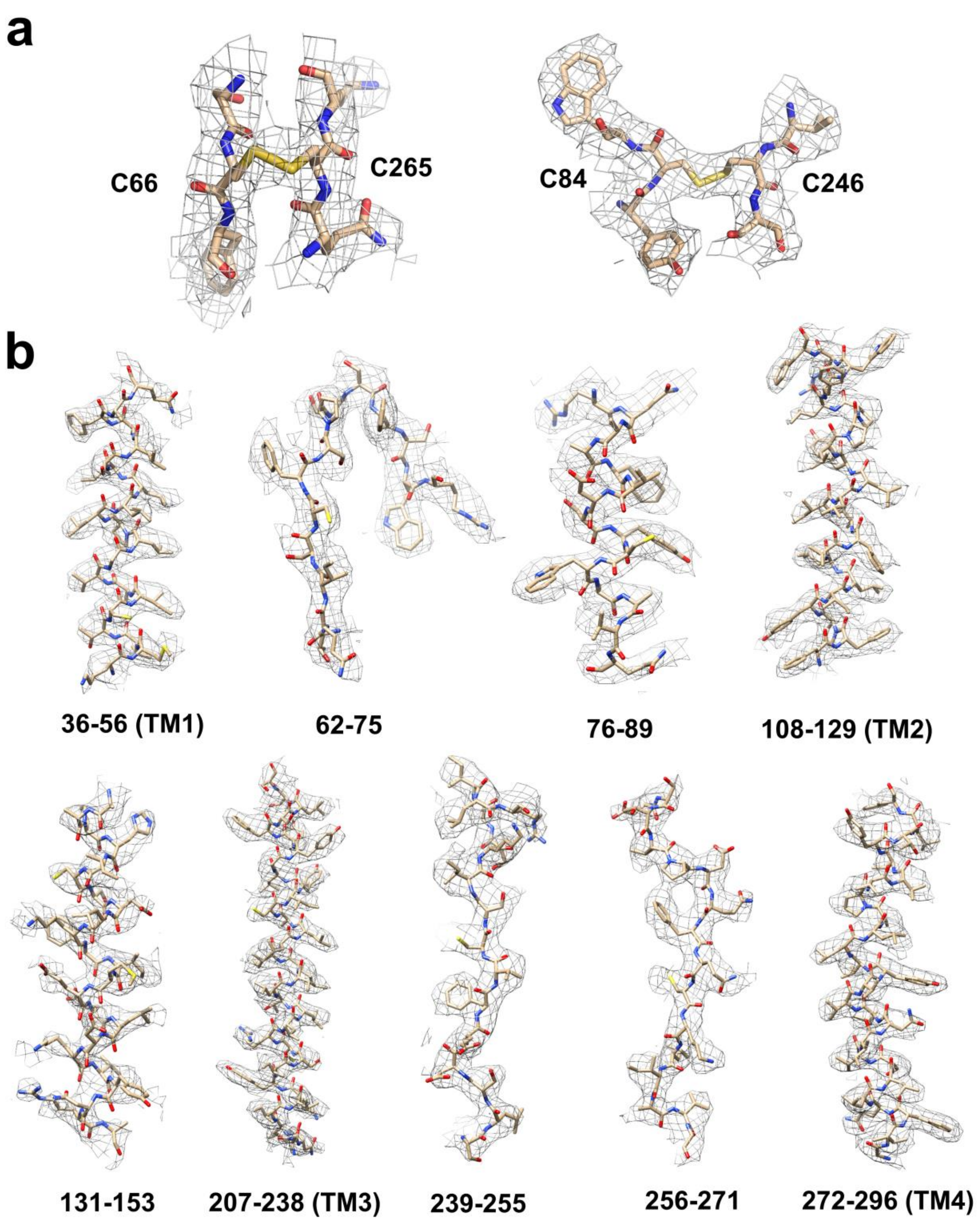

Supplementary Fig. S5. Structural determination and raw cryo-EM densities of hPANX1.

a. Density maps of disulfide bonds in the extracellular region. Corresponding cysteines (C66, C265, C84 and C246) are indicated. The density map is contoured at 8.0 σ.

b. Density maps of representative regions of hPANX1. Stick style atomic models (gold) were fitted into the cryo-EM density maps (gray mesh). The density map is contoured at 8.0 σ.

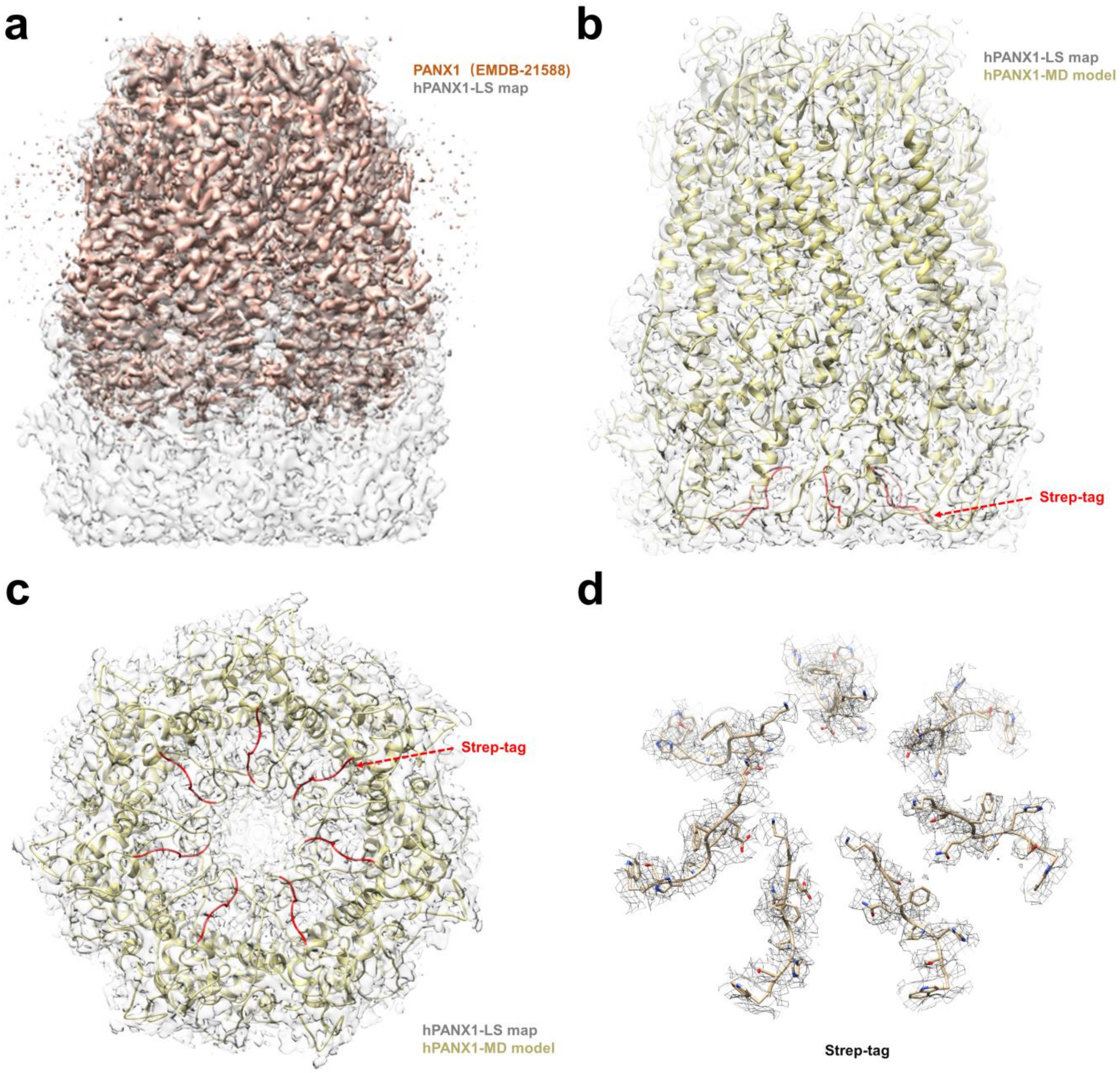

Supplementary Fig. S6. Comparisons between the hPanx1-LS map and other resolved maps.

a. Map comparison between the current hPANX1-LS map (gray) and the reported PANX1 map (EMDB-21588, orange).

b. Schematic representation of hPANX1-MD model in gray docked into the hPANX1-LS map from a side view. The strep-tag location was highlighted in red.

c. Schematic representation of hPANX1-MD model in gray docked into the hPANX1-LS map from a bottom view.

d. The EM density of the Strep-tag (173-180) with a contour level of 4σ.

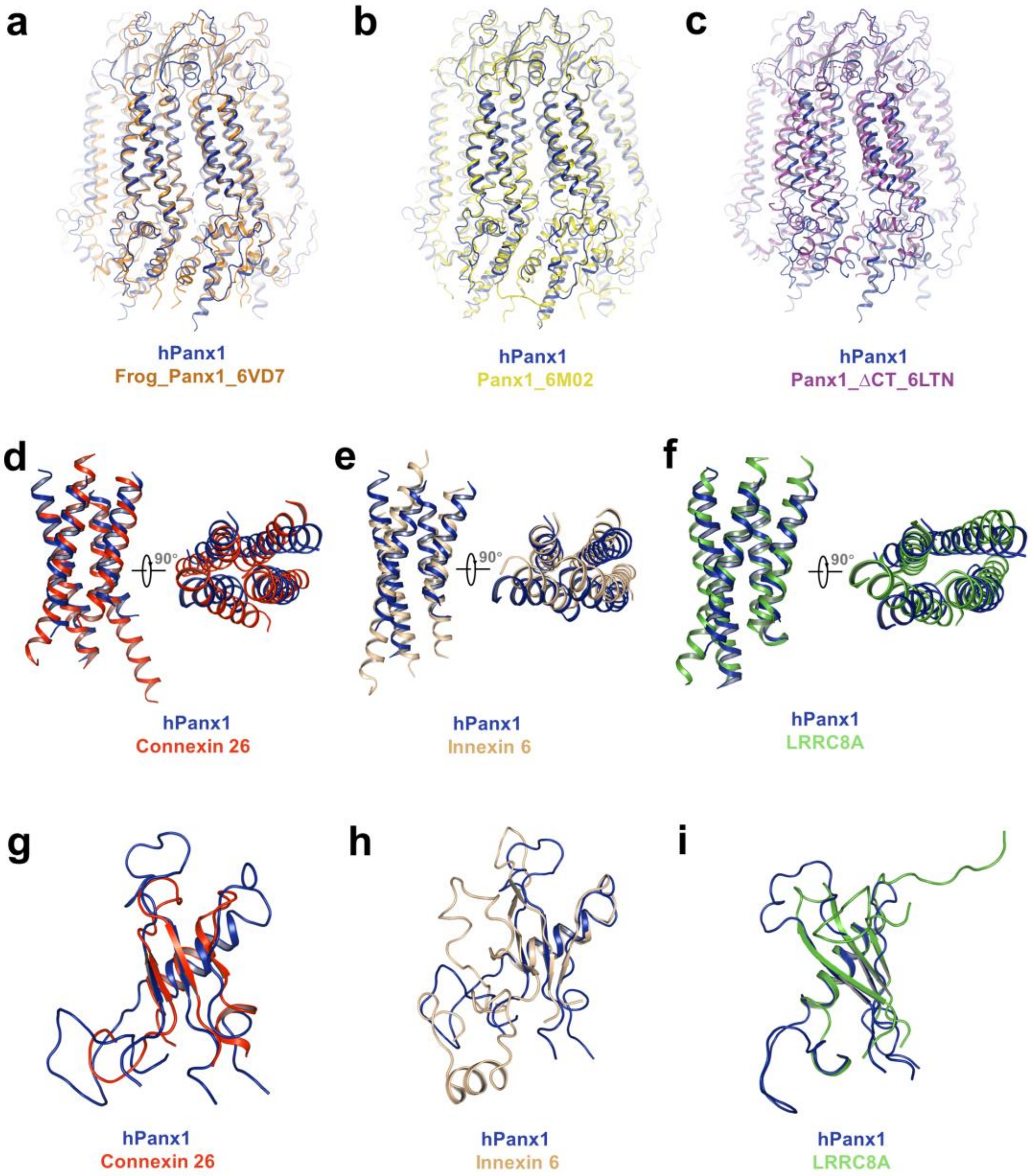

Supplementary Fig. S7. Structure comparisons between hPANX1 and other homologous proteins

a-c: Comparisons between the current hPANX1 (blue) and frog PANX1 (gold, PDB code 6VD7), human PANX1 (yellow, PDB code 6M02), human PANX1 ΔCT (purple, PDB code 6LTN) with a side view.

d-f: Superposition of the transmembrane region between hPANX1 (blue) and Connexin26 (red, PDB code 2ZW3), Innexin6 (brown, PDB code 5H1Q), LRRC8A (green, PDB code 6G9O) with two different views, aligned by TM1 and TM2 helices.

g-i: Superposition of the extracellular region between hPANX1 (blue) and Connexin26 (red, PDB code 2ZW3), Innexin6 (brown, PDB code 5H1Q), LRRC8A (green, PDB code 6G9O).

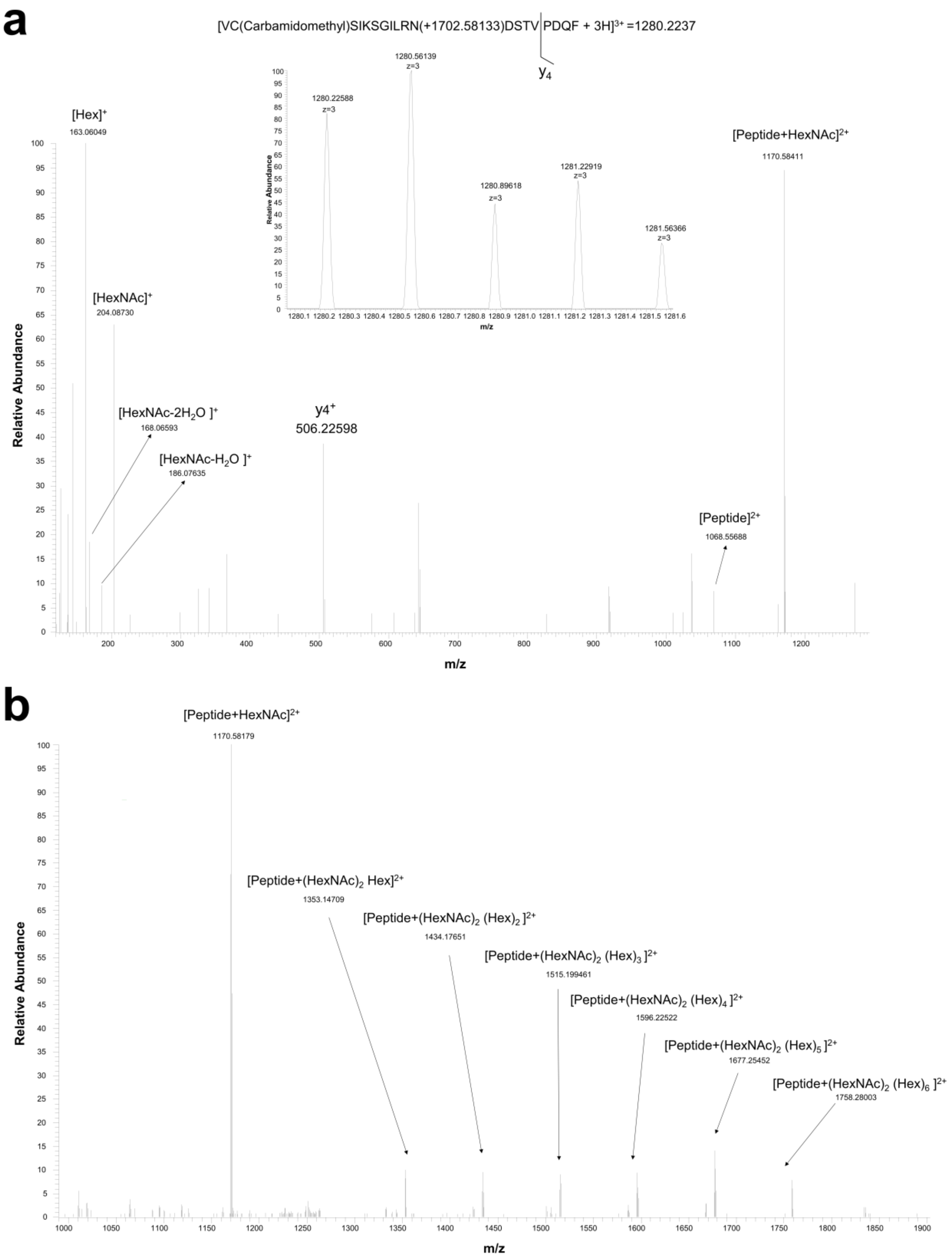

Supplementary Fig. S8. Mass spectrometry of PANX1 glycosylation modification

a. Higher-energy collisional dissociation (HCD) analysis of PANX1 glycosylation modification.

b. Collision-induced dissociation (CID) analysis of PANX1 glycosylation modification.

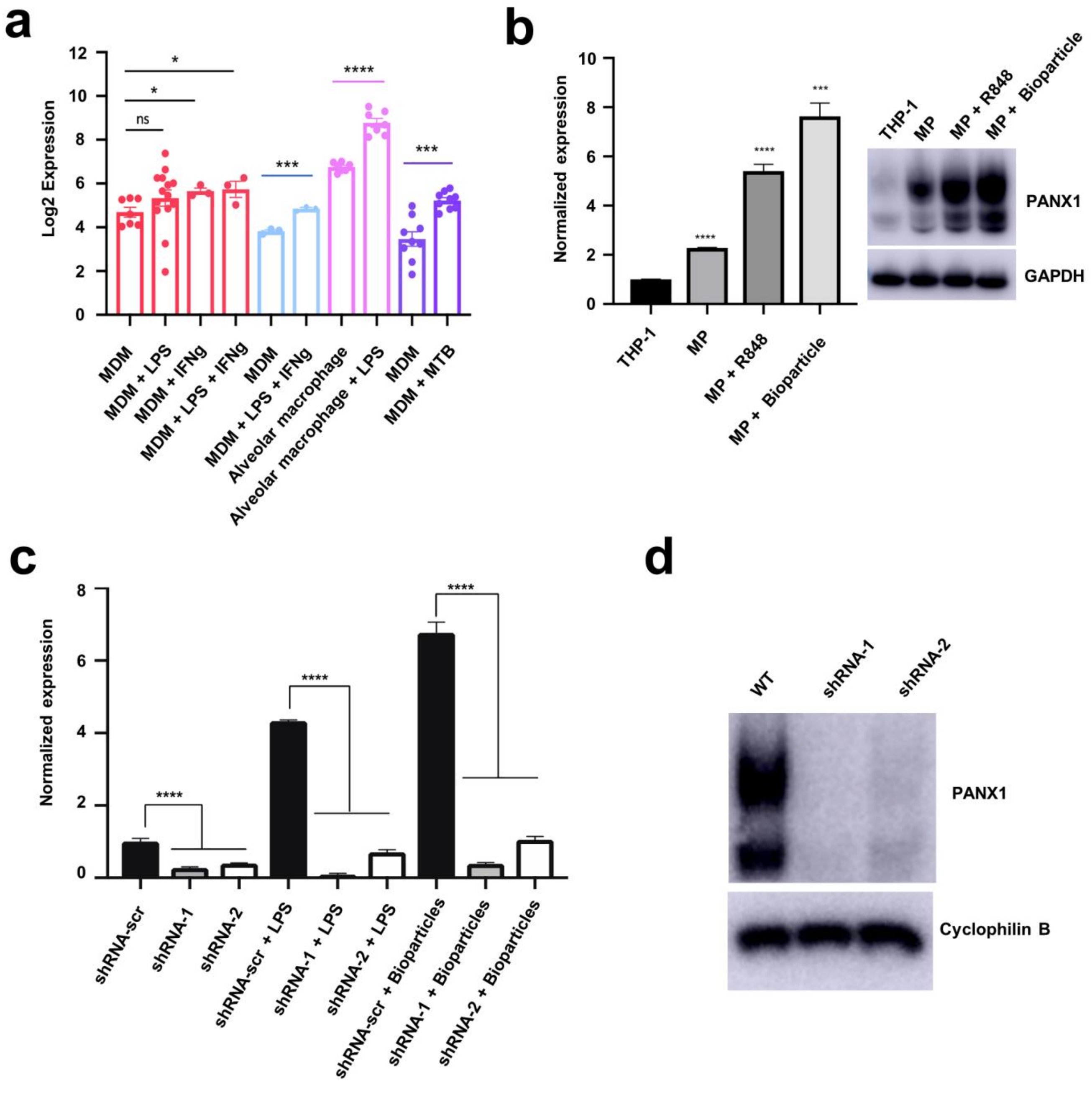

Supplementary Fig. S9. Expression of PANX1 induced by diverse stimulations and establishment of hPANX1 knock-down THP-1-derived macrophage model

a. PANX1 expression data analysis in the Stemformatics database. Four datasets were used here. Red bars show monocyte-derived macrophages (MDM) treated with/without lipopolysaccharide (LPS) and interferon-gamma (IFNg) for 72 hours. Blue bars show transcriptomic profiling of macrophage cells used in disease modeling. Pink bars show alveolar macrophage gene expression profile after LPS stimulation. Purple bars show expression levels of mycobacterium tuberculosis (MTB) infected macrophages. Y-axis represents Log 2 expression values. Data were presented as

the mean ± s.e.m. *$p < 0.05$, ***$p < 0.001$, ****$p < 0.0001$, NS: not significant (Student's t-test).

b. Left, mRNA expression of hPANX1 in MPs, and MPs treated with R848 (30 μM) or Bioparticles for 24 hours (qRT-PCR data were normalized by undifferentiated THP-1 cells); Right, western blot analysis of hPANX1 expression. GAPDH was used as a loading control. Error bars represent s.e.m. for $n = 3$. ***$p < 0.001$, ****$p < 0.0001$ (Student's t-test).

c. shRNA mediated knock-down efficiency in MPs. MPs stimulated by 1 μg/ml LPS or bioparticles for 24 hours. The qRT-PCR data were normalized by the value of shRNA-scr MPs. Error bars represent s.d. for $n = 3$. ****$p < 0.0001$ (one-way ANOVA followed by Dunnett's test).

d. hPANX1 pretein expression levels in WT and hPANX1 knock-down MPs. Cyclophilin B was used as a loading control for the Western blot analysis.

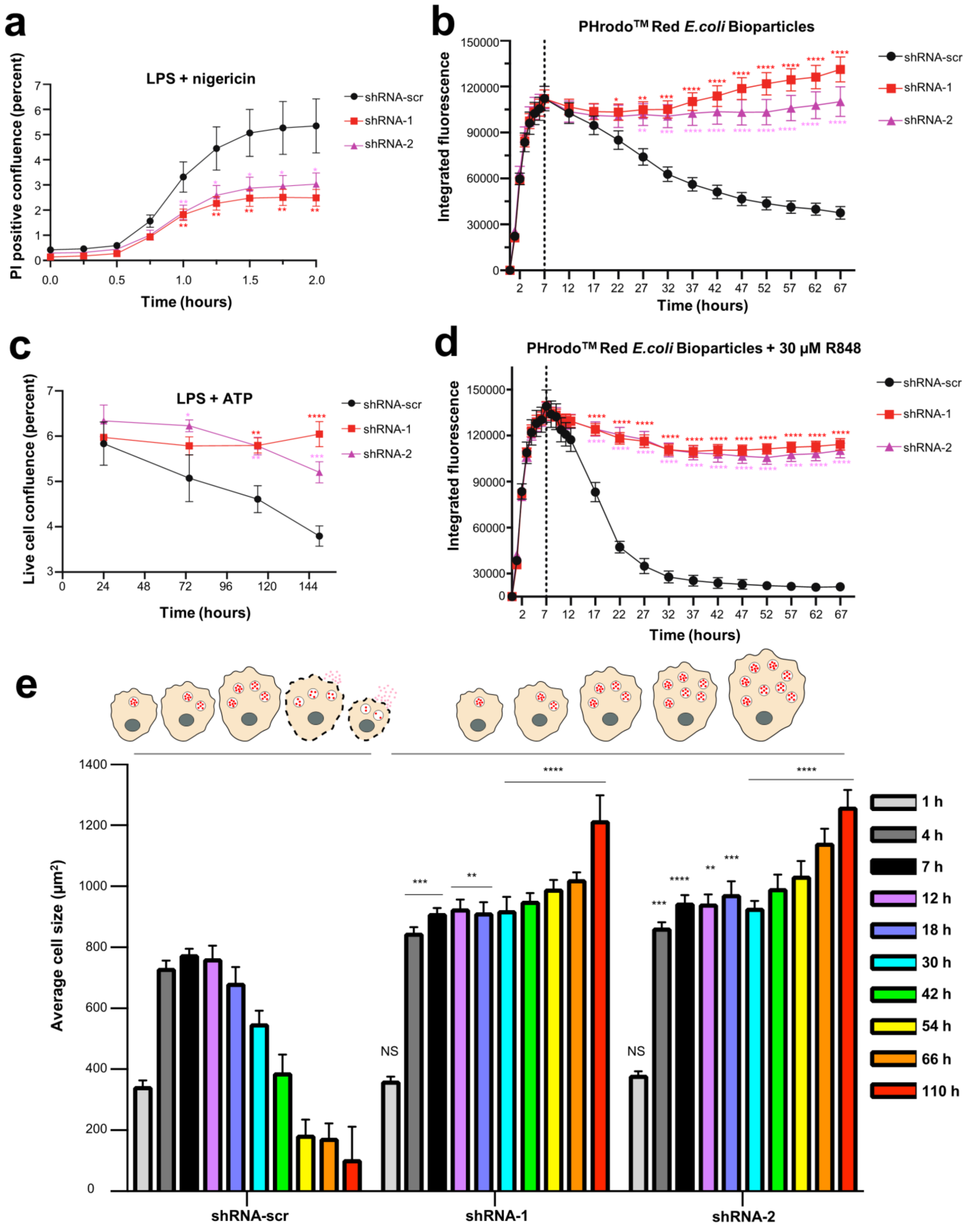

Supplementary Fig. S10. Live cell imaging of hPANX1 knock-down MP model upon pyroptotic stimulations

a. Time course of the confluence of propidium iodide (PI) positive MPs after LPS priming and

nigericin (10 μM) treatment. Live imaging data were collected and analyzed with an IncucyteS3 system. Error bars represent s.d. for $n = 4$. *$p < 0.05$, **$p < 0.01$ (one-way ANOVA followed by Dunnett's test).

b. Time course of fluorescence signal of phagocytosed PHrodo E. coli Bioparticles in MPs stimulated with E. coli bioparticles. Fluorescence images were collected with an IncucyteS3 system every hour. Error bars represent s.d. for $n = 9$. *$p < 0.05$, **$p < 0.01$, ***$p < 0.001$, ****$p < 0.0001$ (one-way ANOVA followed by Dunnett's test).

c. Time course of live cell confluence of MPs after stimulation of LPS (1 μg/ml) and ATP (3 mM). Live imaging data were collected and analyzed with an IncucyteS3 system. Error bars represent s.d. for $n = 4$. *$p < 0.05$, **$p < 0.01$, ***$p < 0.001$, ****$p < 0.0001$ (one-way ANOVA followed by Dunnett's test).

d. Time course of fluorescence signal of phagocytosed PHrodo E. coli Bioparticles in MPs stimulated with E. coli bioparticles and R848. Fluorescence images were collected with an IncucyteS3 system every hour. Error bars represent s.d. for $n = 9$. ****$p < 0.0001$ (one-way ANOVA followed by Dunnett's test).

e. Time course of live cell sizes of MPs stimulated by E. coli bioparticles and R848. Error bars represent s.d.. Around 1,200 cells were counted per time point. **$p < 0.01$, ***$p < 0.001$, ****$p < 0.0001$, NS: not significant (one-way ANOVA followed by Dunnett's test).

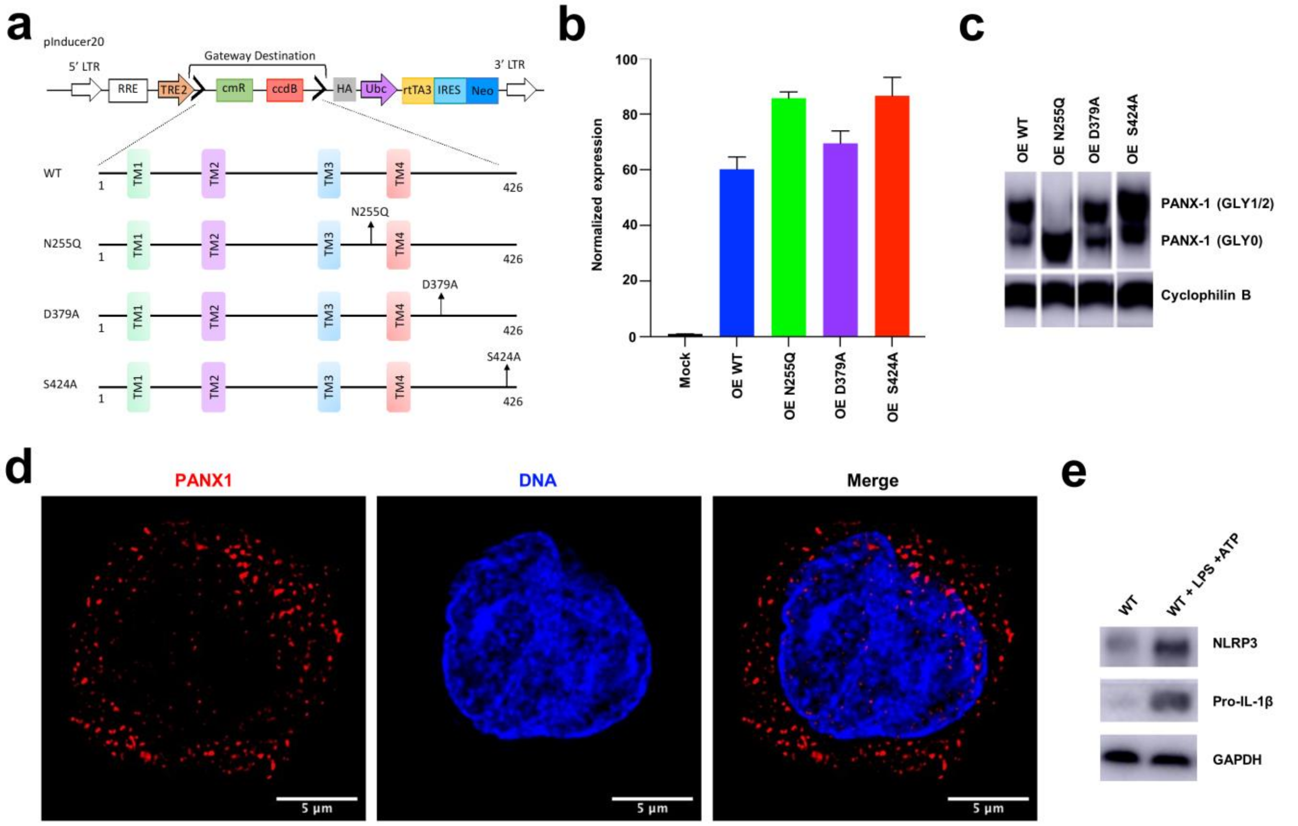

Supplementary Fig. S11. Establishment of WT and mutant hPANX1 overexpression THP-1-derived macrophage model

a. Schematic representation of hPANX1 mutations and reconstruction strategy using gateway cloning to insert hPANX1 sequences into pInducer20 vector, TM: transmembrane domain.

b, c. hPANX1 mRNA and protein expression levels in WT and mutant (D379A and S424A) overexpression MPs. RNA and protein were isolated after 2-day Dox (2 μg/ml) induction. The qRT-PCR data were normalized by the endogenous level of hPANX1 (mock). Cyclophilin B was used as a loading control for the Western blot analysis.

d. Localization of endogenous hPANX1 in MPs. The confocal images were collected using the STED super-resolution mode. Scale bar = 5 μm.

e. Western blot analysis of the NLRP3 and Pro-IL-1β expression levels in endogenous hPANX1MPs with or without treatment of LPS and ATP. GADPH was used as a loading control.

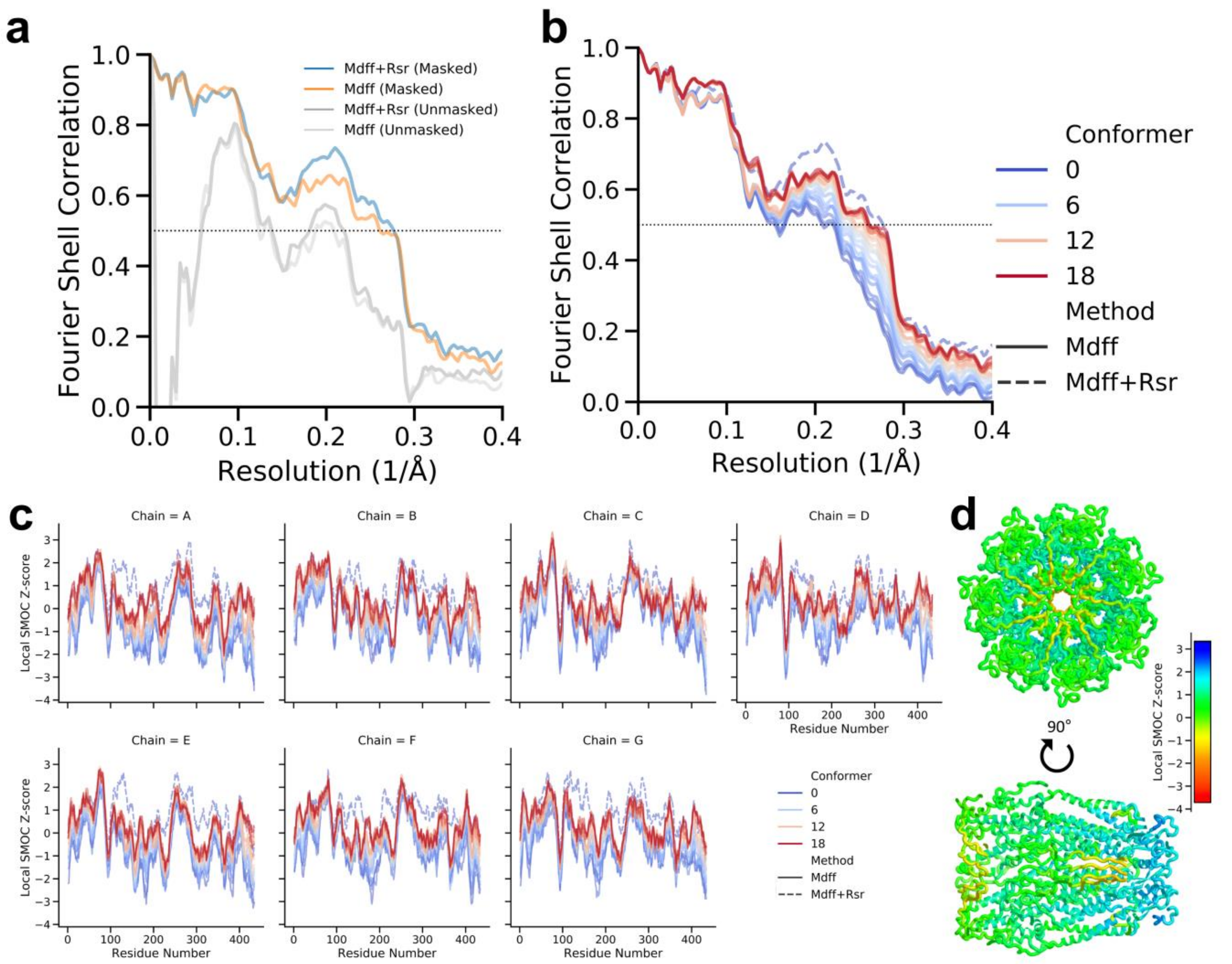

Supplementary Fig. S12. Atom to map resolution analysis of the hPANX1-MD model

a. Atom-To-Map Fourier Shell Correlation (FSC). Two models were compared with the masked and unmasked map. "Mdff" is obtained from the final round of Molecular Dynamics Flexible Fitting (MDFF). "Mdff+Rsr" is the structure obtained after PHENIX Real-Space Refinement (RSR) from "Mdff"; it is also the deposited hPANX1-MD structure. Resolution of the atom-to-map FSC of "Mdff" and "Mdff+Rsr" was determined at the FSC=0.5 line (dotted) to be 3.62 Å and 3.53 Å respectively.

b. Progression of the FSC curve within the eighteen rounds of MDFF fitting. Incremental improvements in resolution from 4.94 Å to 3.62 Å were observed in consecutive rounds of fitting (solid lines ranging from blue to red). FSC progression of the Mdff structures (solid line) is compared to the final "Mdff+Rsr" structure (dashed line).

c. Local resolution analysis using SMOC Z-score within the eighteen rounds of MDFF fitting. Each chain was analyzed separately. Substantial improvement of fitting is achieved during MDFF (solid

lines ranging from blue to red) in the N-/C-termini (residue 2-31 and residue 365-434), regions between TM2/3 (residue 150-216) and regions succeeding TM4 (residue 296-334). SMOC Z-score for the final “Mdff+Rsr” structure is also provided as a reference (dashed line).

d. Projection of SMOC Z-score on “Mdff+Rsr” model. SMOC Z-score is indicated by color.

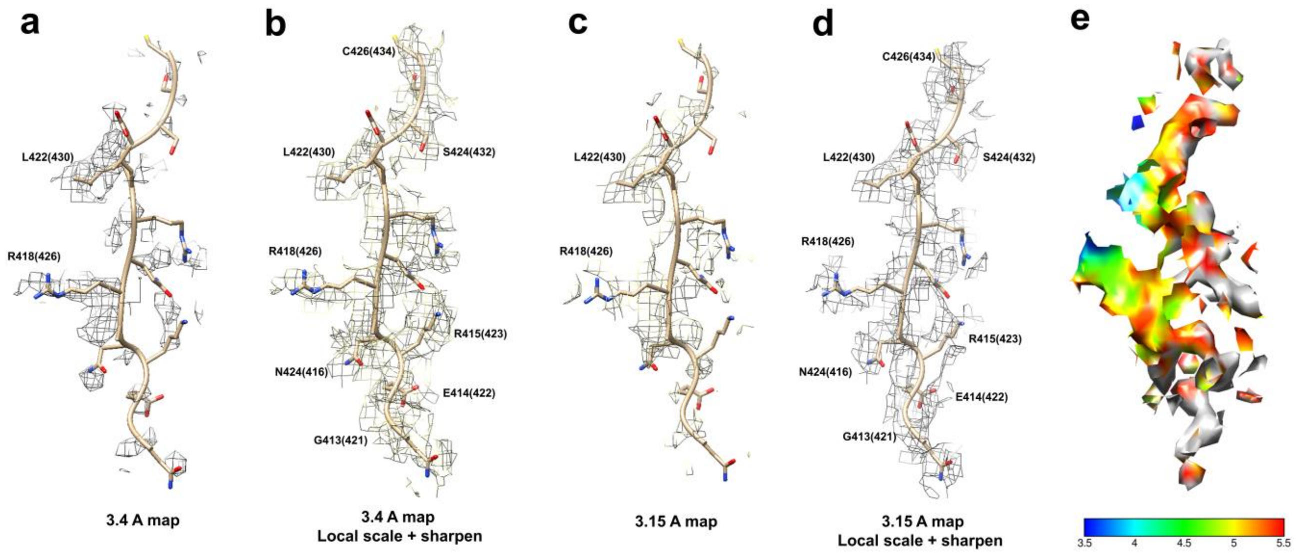

Supplementary Fig. S13. CTL density improvement by local scale and sharpen algorithm.

a. CTL density map of hPANX1 from the 3.4 Å map by RELION software. Corresponding residues (L422 and R418) were labelled.

b. The improvement of CTL density map from the 3.4 Å map after local scale and sharpen algorithm. Representative residues were labelled.

c. CTL density map of hPANX1 from the 3.15 Å map by cryoSPARC software. Corresponding residues (L422 and R418) were labelled.

d. The improvement of CTL density map from the 3.15 Å map after local scale and sharpen algorithm. Representative residues were labelled.

e. Local resolution map of the CTL region density map.

a

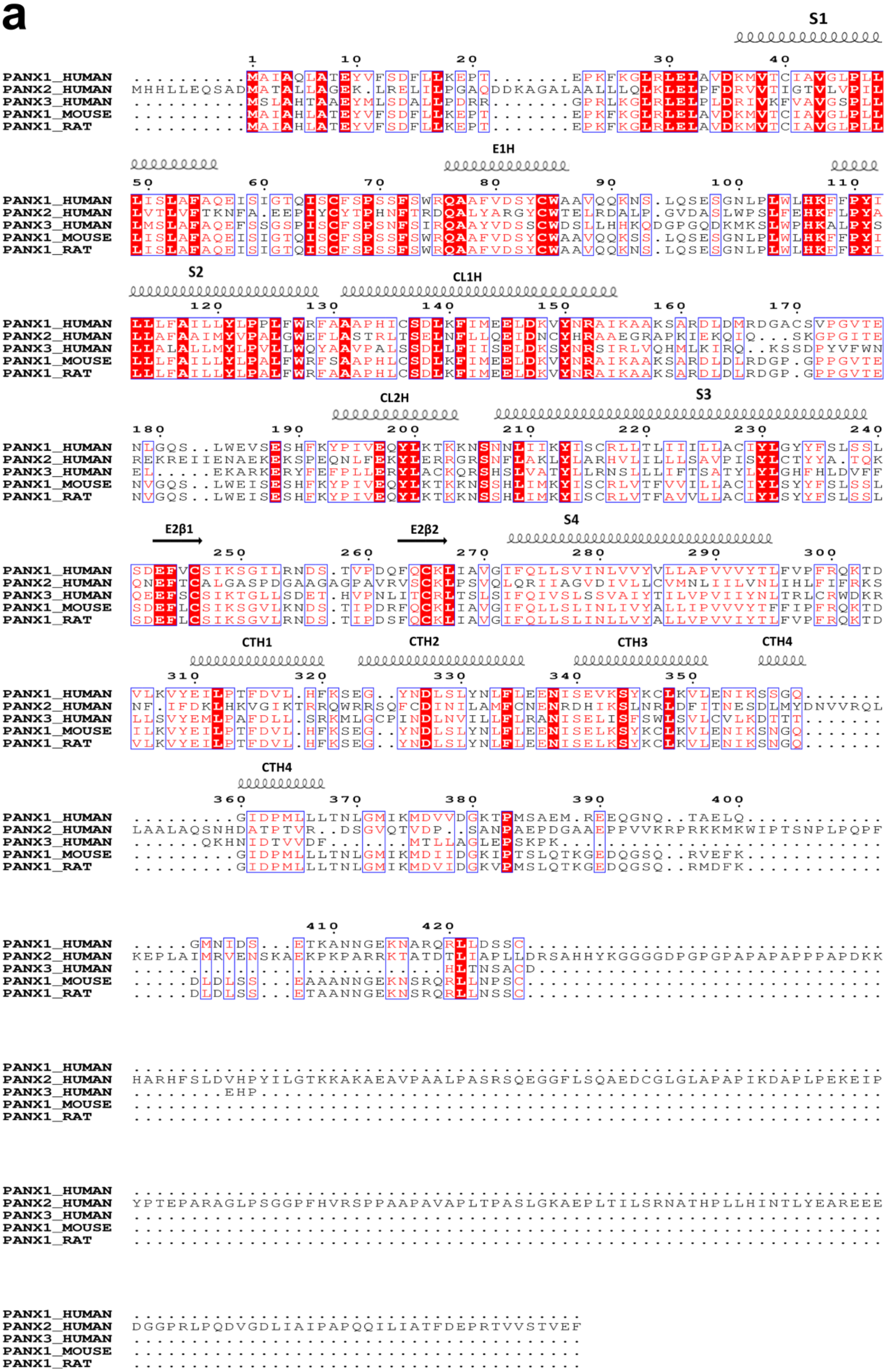
S1
E1H
S2
CL1H
CL2H
S3
E2β1
E2β2
S4
CTH1
CTH2
CTH3
CTH4
PANX1_HUMAN
PANX2_HUMAN
PANX3_HUMAN
PANX1_MOUSE
PANX1_RAT

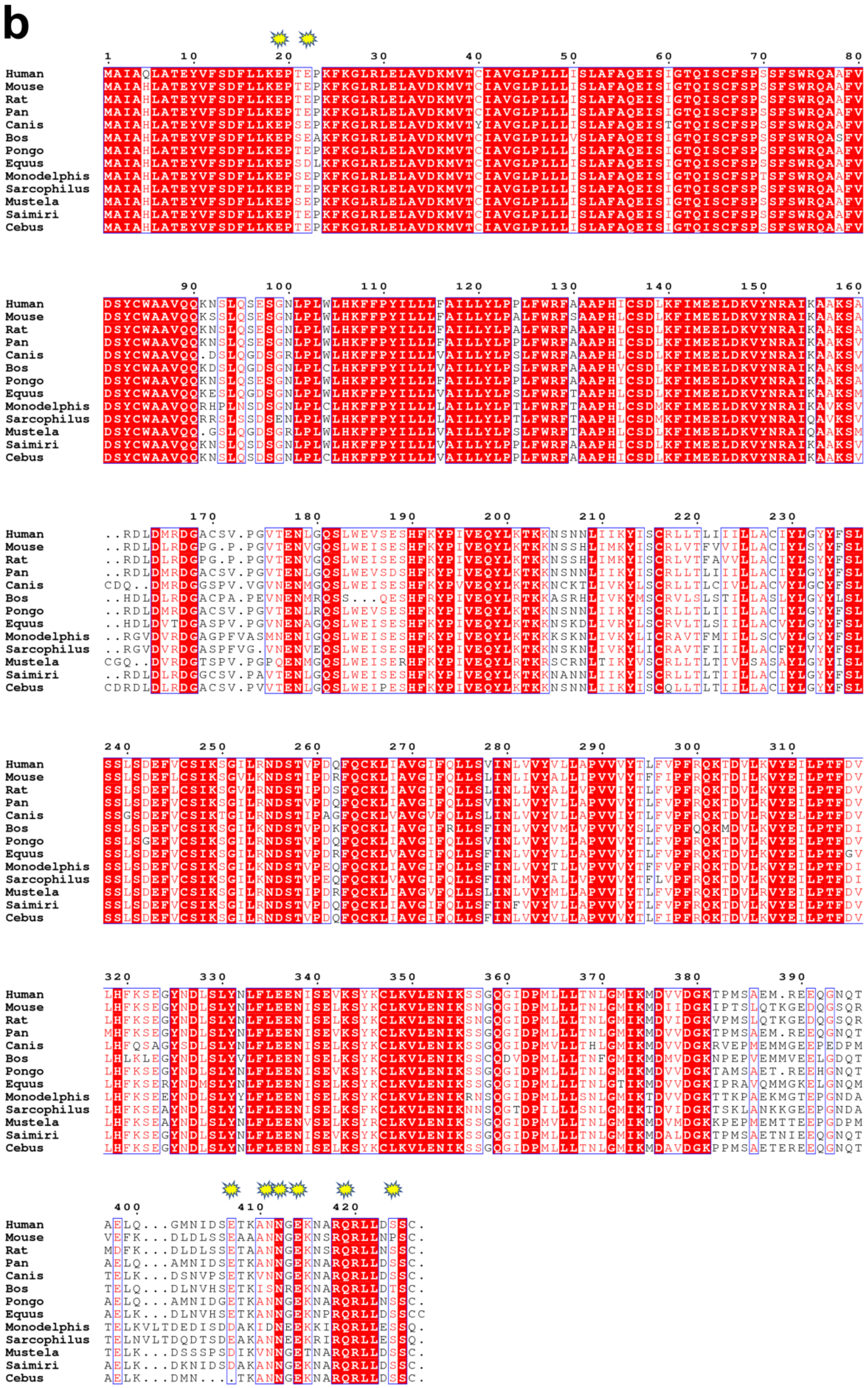
b
Human
Mouse
Rat
Pan
Canis
Bos
Pongo
Equus
Monodelphis
Sarcophilus
Mustela
Saimiri
Cebus

Supplementary Fig. S14. Sequence alignment of Panenxin proteins in different species.

a. Sequence alignment of human PANX1, human PAXN2, human PANX3, mouse PANX1, and rat PANX1 using ESPript3. Secondary structure elements are indicated above the sequence, shown as arrow (β sheet) and helix (α helix).

b. Sequence alignment of PANX1 among mammals using ESPript3. Conserved residues are highlighted and labelled by star marker.

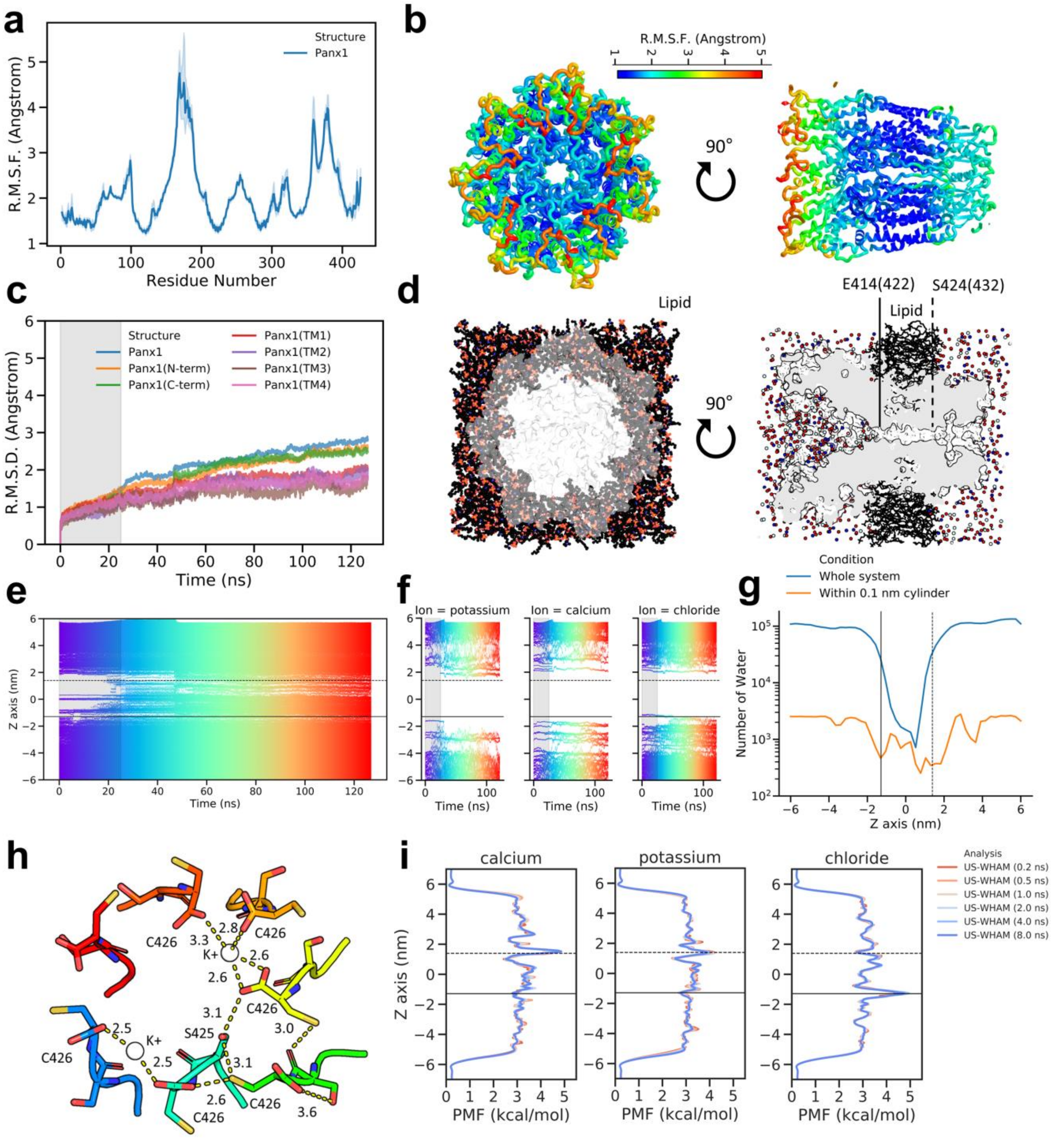

Supplementary Fig. S15. Analyses on molecular dynamics (MD) simulations

a. Plot of average Cα Root-mean-squared fluctuation (R.M.S.F.) during the production phase of the molecular dynamic simulations for PANX1. A 95 % confidence interval obtained by bootstrapping MD frames is shown in shade. Left, front view along z-axis. Right, clipped side view of the system.

b. R.M.S.F value projected on tube representation of the protein backbone. Values corresponding to figure (a) are indicated by color (1.0 –2.0 Å, blue–cyan; 2.0–3.0 Å, green; 3.0–4.0 Å, yellow-orange; 4.0–5.0 Å, orange-red).

c. Root-mean-squared distance (R.M.S.D.) analysis of molecular dynamics simulations preceding umbrella sampling. Cα of the experimental structure (with Strep-Tag excluded) was used as reference for the calculation. Period referring to a 25 ns equilibration phase in indicated with a grey background; 100ns production run in white. R.M.S.D. value of the overall structure is shown in blue. Separate analysis for N-terminus (orange), C-terminus (green), TM1 (red), TM2 (purple), TM3 (brown) and TM4 (pink) are also shown.

d. Snapshot of PANX1 molecular dynamics simulation after equilibration. Left, front view along z-axis showing the PANX1 channel (grey shades) embedded in the POPC lipid bilayer (sticks; carbon in black; oxygen in red, phosphor in orange). Right, clipped side view of the system, potassium K^+ (cyan), and calcium Ca^{2+} (blue) and chloride Cl^- (red) ions are further indicated in spheres. Hydrogens, Water molecules are not shown.

e-f. Positions of water molecules and ions along the z-axis as a function of time. Two black horizontal lines are used to indicate the average atomic position of E414 (solid) and S424 (dash). Each water molecule is marked by a point colored in rainbow along the time axis. Period referring to a 25ns equilibration phase in indicated with a grey background; 100ns production run in white. Intermittent unmarked regions can be found in the production run around E414 and S424, indicating transient but not prolonged dewetting.

g. The total number of water molecules across 100 snapshots taken uniformly from the 100ns production run as a function of average position along z-axis. Note the use of a logarithmic scale for the vertical axis. Aside from counting across the whole system (blue), we also count water molecules from within a cylinder of radius 1nm enclosing the pore axis (orange). Two black vertical lines are used to indicate the average atomic position of E414 (solid) and S424 (dash).

h. Transient cation binding sites at terminal C426 illustrated by a MD snapshot. S425-C426 are shown

in stick. Hydrogen or electrostatic bonds are shown as yellow dash lines labelled with distance in angstrom.

i. Convergence of Potential of Mean Force (PMF) Profiles for potassium (left), calcium (middle) and chloride (right). We show the progression of the profile with respect to increasingly lengthier umbrella sampling ranging from 0.2 ns to 8.0 ns indicated by color from red to blue. Two black vertical lines are used to indicate the average atomic position of E414 (solid) and S424 (dash).

Supplementary Movies

Supplementary Movie S1-3: Scrambled shRNA transduced MPs dying after engulfment of *E. coli* bioparticles. Images were collected 10 hours after phagocytosis and taken every hour for 3 days. Video was generated at a speed of 3 frames per second.

Supplementary Movie S4-6: Panx1 shRNA-1 transduced MPs persisting and becoming filled with growing vacuoles after engulfment of *E. coli* bioparticles. Images were collected 10 hours after phagocytosis and taken every hour for 3 days. Video was generated at a speed of 3 frames per second.

Supplementary Movie S7-9: Panx1 shRNA-2 transduced MPs persisting and becoming filled with growing vacuoles after engulfment of *E. coli* bioparticles. Images were collected 10 hours after phagocytosis and taken every hour for 3 days. Video was generated at a speed of 3 frames per second.

Supplementary Movie S10: Mean-shift-algorithm applied to visualize the local dense points (white trails) of a locally sharpened density map followed by superposition with the full-length hPanx1-MD model; C terminal constriction residues were labelled in the video.

Supplementary Table S1 | Cryo-EM data collection, refinement and validation statistics

	hPanx1_MD_model<br>(EMDB-30880, 30881)<br>(PDB 7DWB)
Data collection and processing	
Magnification	130,000
Voltage (kV)	300
Electron exposure (e^{-}/Å^{2})	50
Defocus range (μm)	-1.3 ~ -2.3
Pixel size (Å)	1.08
Software	RELION-3.0/cryoSPARC
Symmetry imposed	C7
Initial particle images (no.)	1662,000
Final particles images (no.)	113,000
Map resolution (Å)	3.15
FSC threshold	0.143
Local map resolution range (Å)	4.3-2.3
Refinement	
Software	PHENIX 1.14
Initial model used (PDB code)	
Model resolution (Å)	3.2
FSC threshold	0.5
Map sharpening *B* factor	-221.3
Model composition	
Non-hydrogen atoms	23989
Protein residues	3031
Ligand	0
B factors (Å^{2})	
Protein	126.22
Ligand	0
R.m.s deviations	
Bond length (Å)	0.0039
Bond angles (°)	1.03
Validation	
MolProbity score	1.96
Clashscore	6.40
Poor rotamers (%)	0
Ramachandran plot	
Favored (%)	87.70
Allowed (%)	12.06
Disallowed (%)	0.23
